# Recent trends and economic significance of modified/functionalized biochars for remediation of environmental pollutants

**DOI:** 10.1038/s41598-023-50623-1

**Published:** 2024-01-02

**Authors:** Ghulam Murtaza, Zeeshan Ahmed, Mohammad Valipour, Iftikhar Ali, Muhammad Usman, Rashid Iqbal, Usman Zulfiqar, Muhammad Rizwan, Salman Mahmood, Abd Ullah, Muhammad Arslan, Muhammad Habib ur Rehman, Allah Ditta, Akash Tariq

**Affiliations:** 1 Faculty of Environmental Science and Engineering, Kunming University of Science and Technology, Kunming, 650500 China; 2 Xinjiang Institute of Ecology and Geography, Chinese Academy of Sciences, Urumqi, 830011 Xinjiang China; 3 Xinjiang Institute of Ecology and Geography, Cele National Station of Observation and Research for Desert-Grassland Ecosystems, Chinese Academy of Sciences, Xinjiang, 848300 China; 4 Department of Engineering and Engineering Technology, Metropolitan State University of Denver, Denver, CO 80217 USA; 5 Center for Plant Science and Biodiversity, University of Swat, Charbagh, Pakistan; 6 Department of Botany, Government College University, Katcheri Road, Lahore, 54000 Punjab Pakistan; 7 School of Agriculture and Biology, Shanghai Jiao Tong University, Shanghai, China; 8 Department of Agronomy, Faculty of Agriculture and Environment, The Islamia University of Bahawalpur, Bahawalpur, Pakistan; 9 School of Energy Science and Engineering, Central South University, Changsha, 410011 China; 10 Faculty of Economics and Management, Southwest Forestry, Kunming, Yunnan, 650224 China; 11 Institute of Crop Science and Resource Conservation (INRES), University of Bonn, Bonn, Germany; 12 Department of Seed Science and Technology, Institute of Plant Breeding and Biotechnology (IPBB), MNS-University of Agriculture, Multan, Pakistan; 13 Department of Environmental Sciences, Shaheed Benazir Bhutto University Sheringal Dir (U), KPK, Sheringal, Pakistan; 14 School of Biological Sciences, The University of Western Australia, Perth, WA 6009 Australia

**Keywords:** Environmental sciences, Environmental biotechnology

## Abstract

The pollution of soil and aquatic systems by inorganic and organic chemicals has become a global concern. Economical, eco-friendly, and sustainable solutions are direly required to alleviate the deleterious effects of these chemicals to ensure human well-being and environmental sustainability. In recent decades, biochar has emerged as an efficient material encompassing huge potential to decontaminate a wide range of pollutants from soil and aquatic systems. However, the application of raw biochars for pollutant remediation is confronting a major challenge of not getting the desired decontamination results due to its specific properties. Thus, multiple functionalizing/modification techniques have been introduced to alter the physicochemical and molecular attributes of biochars to increase their efficacy in environmental remediation. This review provides a comprehensive overview of the latest advancements in developing multiple functionalized/modified biochars via biological and other physiochemical techniques. Related mechanisms and further applications of multiple modified biochar in soil and water systems remediation have been discussed and summarized. Furthermore, existing research gaps and challenges are discussed, as well as further study needs are suggested. This work epitomizes the scientific prospects for a complete understanding of employing modified biochar as an efficient candidate for the decontamination of polluted soil and water systems for regenerative development.

## Introduction

**Figure 1:**
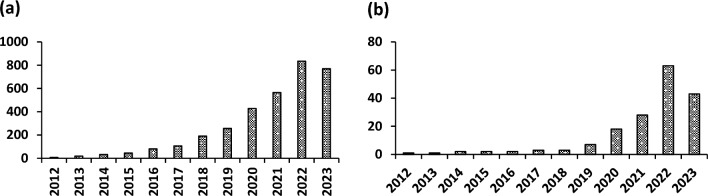
The number of papers (**a**) research + review and (**b**) review articles published in the last 10 years.

Biochar is a carbon-rich material produced from different organic waste feedstocks, such as municipal sewage sludge and agricultural wastes^1^. Biochar gained much attention due to its unique characteristics such as large specific surface area, stable structure, high cation exchange capacity, and carbon content^1,2^. Its significance could be realized by the increasing number of published articles in the last ten years (Fig. 1). Biochar can amend the fertility of the soil and can sequester carbon; hence it can potentially lead to the mitigation of climate change^2–5^. To enhance soil fertility and carbon sequestration potential, biochar improves physical (moisture level, oxygen content, and capacity of water holding), chemical (sequestration of carbon and immobilization of pollutants), and biological (microbe’s abundance, activity, and diversity) properties of soil. It also helps in the removal of various contaminants from soil and water systems^3–6^. Various conventional methods are used to remove organic, inorganic, and other emerging pollutants from the water and soil, such as coagulation/flocculation, chemical precipitation, and biochemical degradation^3^. These methods usually eliminate valuable contaminants from water and soil but have low efficiency with high operational and maintenance costs and massive waste production^2–4^. In contrast, adsorption using agricultural organic wastes is emerging as a cost-effective, user-friendly, and efficient method for removing various impurities from soil and water systems^5^. Adsorption is a key mechanism for biochar to eliminate organic and inorganic pollutants. The adsorption capability of biochar is directly linked to its physicochemical attributes such as functional groups, cation exchange capacity, distribution of pore size, and surface area, however, these attributes vary with the production conditions like nature of biomass utilized for biochar production, pyrolysis temperature, etc.^2,3^ However, pristine biochar due to limited adsorption sites and low surface functionality does not display specific and high nutrient adsorption capability^4^. To enhance the adsorption capacity, biochars are modified/functionalized using multiple-modification agents including alkali, acids, metal oxides, and oxidizing agents, which manifest improved surface properties and novel structures after treatment^1,4,5^. Compared to pristine biochar, modified/functionalized biochar with enlarged surface area and abundant functional groups presents a new type of carbon-based material with enhanced adsorption potential for pollutants in water and soil systems^3,5^. Generally, functionalization techniques for biochar can be considered into three main modification types such as biological, chemical, and physical^6^. Chemical modification techniques include oxidizing treatment, soaking with acid and base, magnetization, loading of carbon nanomaterials, doping with clay minerals, organic surfactants, non-metallic elements, and layered double hydroxides^7,8^. These modifications not only improve the biochar's physical attributes but also influence its chemical characteristics such as surface functional groups, elemental distribution, zeta potential, electron transfer capacity, and cation exchange capacity due to their impact on porosity and enrichment of biochar surface with O-containing functional groups, especially carboxyl ones^6,7^. Physical modification such as activation by CO_2_/steam and microwave, and ball milling improves the particle size, pore structure, functional groups, and surface area of biochar^8,9^. It provides advantages over chemical techniques being less polluted in nature and economically more viable for biochar fabrication^8^. Moreover, chars can be functionalized via biological technique; which carries advantage of various microbes and biological-linked methods, and further assists in the elimination of toxic contaminants^9–11^. There have been few reviews focusing on diverse applications of biochar such as soil fertility and quality improvement, catalysis, and aqueous pollutant removal^10^. So far, various studies emphasize more on applying raw and modified biochar to eliminate pollutants from the water system^4,5,7,8,12,13^. Nonetheless, a comprehensive study including the use of multiple-functionalized biochar-based adsorbents in the removal of pollutants from soil and aquatic systems has scarcely been described. Furthermore, the compiled knowledge of multiple functionalization techniques for char/adsorbents, for example, doping of non-metallic heteroatom is scanty. Aiming to describe a thorough analysis of multiple-functionalized biochars for the remediation of environmental impurities, based on recently published literature, this updated study exhaustively outlined the novel approaches in multiple functionalized techniques for the biochars. Moreover, the reusability of modified/functionalized biochar as well as the economic perspective of the biochar production and application as compared to other expensive sorbents like activated carbon has also been discussed. This review could be helpful in the large-scale preparation and application of modified/functionalized biochars for managing polluted soil and aquatic systems and may ensure the sustainable protection of the environment.

## Various modification techniques for the preparation of functionalized biochars and effects on the water system

**Figure 2:**
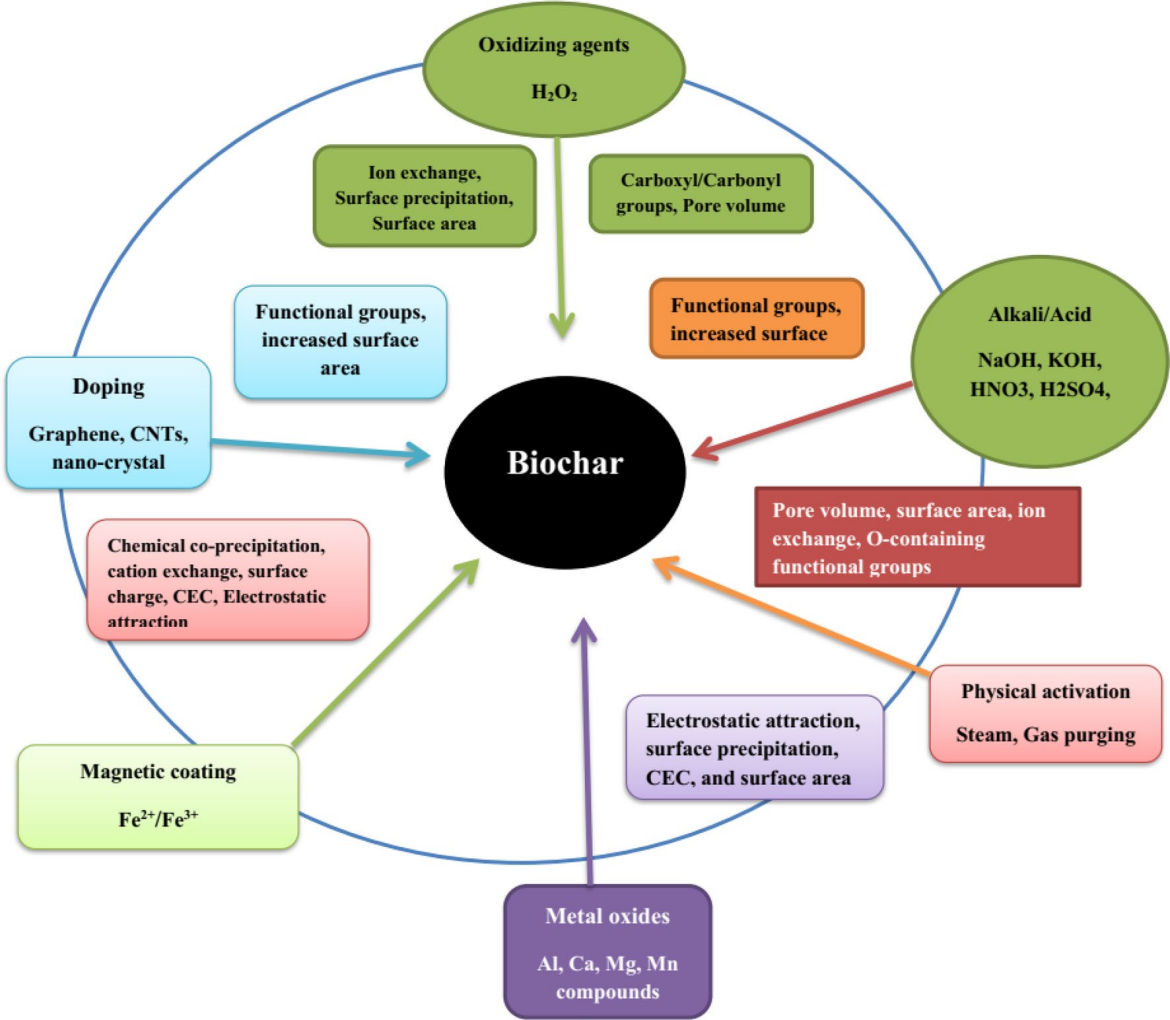
Schematic illustrations of biochar modifications.

Accessible modification techniques have been scrutinized in the published literature and are summed up in (Table 1) which can be distributed into 4 major classes, including physical and chemical modifications, magnetic modifications, and soaking with minerals (Fig. 2). Changes in biochar physiochemical properties after multiple modifications are detailed in (Table 2).

**Table 1:** Various modification approaches of biochars, production temperature, pollutant removals from the water and soil systems, mechanisms, and their applications.

Biochar Feedstock	Pyrolysis temperature (°C)	Modification method	Target contaminant	Decontamination status	Mechanism involved	References
Chemical modification						
Peanut hull	300	H_2_O_2_ treatment	Cd, Ni, Cu, and Pb	increased Pb sorption from 0.88 to 22.82 mg g^−1^, which was higher than commercial AC	Increased oxygen-rich functional groups on the biochar surfaces	^11^
Bamboo	550	Chemical oxidation (NaOH, HNO_3_)	Furfural	suppressed the sorption of furfural	A substantial amount of acidic functional groups on the adsorbent surface. Contrastingly, heat and NaOH modifications raised the basicity of adsorbent	^12^
Municipal waste	400–600	KOH modification	Arsenic pentoxide	Increased 1.3 times adsorption rate than un-treated biochar	Enhance SSA and alter the porous structure, particularly functional groups on the surface of the modified adsorbent	^13^
Pine-chips	300	NaOH treatment	Ibuprofen, Naproxen and Diclofenac	showed greater sorption efficiency	Large amounts of oxygen-enrich functional groups introduced on the surface of treated biochar	^15^
Rice husk	400, 500	Treated by H_2_SO_4_ and KOH	Tetracycline	Shown better adsorption efficiency (58.8 mg g^−1^) compared to other biochars	owned larger SA than those of acidic-modified and pristine biochars	^16^
Sawdust	500	Amino-treated	Copper (Cu)	Improved the sorption up to 5-folds and 8-folds for fixed-bed and batch experiments	Amino moiety strongly complexes with heavy metals because of the high stability constants of metal complexes	^17^
Rice husk	400, 500	Methanol-treated	Tetracycline	Almost 45% heightening of removal capacity in 12 h and 17% at equilibrium	Due to alteration in oxygen-comprising functional groups	^9^
Buttonwood waste	400	Modified by (Mg(OH)_2_)	Fe^2+^	Greater removal capacities for treated biochar (84–99%) than by un-treated biochar (38–97%)	Mineral constituents e.g., silicate Mg(OH)_2_ and calcite in the biochars stimulate the oxidation of Fe^2+^ and form a precipitate of Fe^3+^ hydroxides	^18^
Rice husk	450 and 500	Polyethylenimine treatment	Chromium	Highest removal capability of (435 mg g^−1^), it was better than Un-treated biochar (23.09 mg g^−1^)	The appearance of the amino- group stimulates the chemical reduction of chromium and enhances the removal capacity	^19^
Walnut-chips	600	Carbon nanotube-coating	Methylene blue	Maximum removal capacity among all contaminants	Coated biochar has well thermal stability, greater SA, and higher pore volume	^12^
Rice husk and fruit branches	600	Ferric coated	As (III) and As (V)	Enhancement of removal capacities	Interactions with FeOH_2_ and FeOH groups	^20^
Sawdust and pine tree	550	H_3_PO_4_ modification	Fluoride	Substantial increase in removal performance modification	Increasing Fluoride sorption resulting from chemistry reaction and increased SSA	^21^
Rice husk	600	Coated with silica	Pb	Improvement of removal capacities	A larger SSA observed after coating	^7^
Wheat straw	450	Coated with Fecl_3_ and treated by HCL	phosphate and nitrate	Substantial increase of removal after HCl treatment and coating with Fecl_3_	–	^4^
Wheat straw	300, 700	Acid activation	Sulfamethazine	Noteworthy increase in SA and enhancement in the removal of sulfamethazine	–	^22^
Bagasse	600	Modified by carbon nanotube	Sulfapyridine and Pb	Maximum sorption capacity observed	–	^13^
Bamboo hardwood	550	NaOH modification	Cd	Highest cadmium sorption capacity	NaOH-treated adsorbent has more roughness compared to un-treated biochar	^23^
Cow manure and wheat straw	450	HNO_3_ treatment	U(VI)	Showed the highest sorption capacities after modification, it was higher than un-modified biochar, Highest removal capacity by the treated wheat straw adsorbent exhibited an enhancement of 40 times	Due to a large number of surface COO groups, a great negative surface charge	^18^
Swine manure and rice straw	700	H_3_PO_4_ modification	Tetracycline	Increased the TC removal capacity	Enhancement of the SSA, higher micropore, and total pore after treatment	^15^
Poplar chips	550	AlCl_3_-modification	PO_4_^3−^, NO^3-^	PO_4_^3−^, NO^3−^ removal significantly enhanced on Al-treated biochar	The surface area markedly improved with the Al content of the adsorbent. The C content of Al-treated biochar greatly decreased than pristine biochar	^3^
Dairy manure	300	NaOH-modification	Cd, Pb	The highest removal capacities were 68.08 and 175.53 mg g^−1^ for Cd and Pb respectively. The sorption capacities of dairy manure biochar for Cd and Pb improved after modification	NaOH modification increased the SSA, amount of O-enrich functional group, and ion-exchange capacity of biochar	^8^
Coconut shell	800	HCl + ultra-sonication	Zn, Ni, and Cd	Modified biochar showed the highest sorption capacities for heavy metals	Modified-biochar improved surface functional groups	^15^
Corn straw	500	KOH	Atrazine, Hg(II)	The sorption capacity of treated biochar for Hg (II) enhanced by 76.95%, while that for atrazine enhanced by 38.66%	After modification enhanced SA which was 59.23 m^2^ g^−1^	^23^
Auricularia auricular dreg	400	Cetyl trimethyl ammonium bromide	Cr (IV)	The removal rate increased by 40 times more as compared to un-treated biochar	The number of micropores and mesoporous in the unit area enhanced, After treatment, the SA enhanced by 6.1% and the average pore diameter increased by 16.5%	^24^
Seaweed	200	KOH	V(V)	12 mg g^−1^ sorption capacity noticed	Complexation, electrostatic interaction and pore diffusion	^25^
Rice straw	400	β-cyclodextrin and HCl	Pb^2+^	130 mg g^−1^ sorption capacity found was higher than unmodified biochar	Complexation, ion exchange, and physisorption	^29^
Horse manure	500	Bismuth(III) nitrate	U(VI)	516 mg g^−1^ adsorption capacity found was higher than un-modified biochar	Reductive reaction, ion exchange, and precipitation	^11^
Physical modification						
Bur cucumber	300, 700	Steam activation	Sulfamethazine	Around 55% enhancement in removal capacity	–	^10^
Whitewood	550	Steam activation	Emission of CH_4_	Suppress CH_4_ emission	–	^2^
Maize stover	350	Steam activation	Emission of N_2_O	Suppress N_2_O emission	–	^26^
Tea waste, soybean straw, bagasse, and shrub	300, 700	Steam activation	Sulfamethazine	Maximum sulfamethazine sorption among all the biochars	Due to its higher SA and pore volume	^27^
Guayule, corn stover and cob, switchgrass, alfalfa stems, and chicken manure	500	Steam activation	Cu	Highest sorption capacities observed	Largest SSA and porous structure	^28^
Cornstalk	500, 900	CO_2_/NH_3_ Modification	CO_2_	–	NH_3_ reacts with the biochar surface, introducing the nitrogen functional groups; CO_2_ modification forms more micropore	^30^
Black spruce	454, 900	Steam activation	Sulfur dioxide	The sorption capacity of sulfur dioxide was found higher (76 mg g^−1^)	Surface area (590 m^2^ g^−1^) and pore volume increased	^31^
Canola straw	700	Steam modification	Pb (II)	Removal capacity observed (195 mg g^−1^)	Due to its higher SA and pore volume	^30^
Rice straw	800	Steam activation	Naphthalene	The sorption rate was noticed at 76%	Higher surface area (106 m^2^ g^−1^) and a large amount of surface functional groups	^32^
Poplar wood	300	Ball milling	Mercury	Sorption capacity was 320 mg g^−1^	Surface area and pore structure improved	^33^
Soybean straw	800	Steam activation	Zn^2+^, Ni^2+^, Cd^2+^, and Cu^2+^	Removal capacity 27.8, 30, 21,95.7 mg g^-1^ for Zn^2+^, Ni^2+^, Cd^2+^, Cu^2+^	Higher surface area (793 m^2^ g^−1^) and average pore diameter enhanced	^34^
Bamboo	500	Activation by steam	Tetracycline and Copper (II)	Adsorption capacity 0.22 and 5.03 mmol g^−1^ tetracycline and Copper (II), respectively	Due to changes in oxygen-enrich functional groups	^27^
Mushroom	800	Steam activation	Crystal violet	1057 mg g^−1^ adsorption capacity found	Higher surface area (332 m^2^ g^−1^)	^24^
Invasive plants	700	Steam modification	Sulfamethazine	37.7 mg g^−1^ adsorption capacity observed	Because of higher SA and pore volume	^22^
Dendro	700	Ball milling	Cadmium and chromium	Sorption capacity for chromium 922 mg g^−1^ and cadmium 7.46 mg g^−1^	Improved pore structure after modification	^30^
Tea waste	700	Steam activation	Sulfamethazine	33.81 mg g^−1^ adsorption capacity noticed	Higher surface area (576.9 m^2^ g^−1^) and a large amount of surface functional groups	^35^
Hickory chip	600	Ball milling	Reactive red	34.80 mg g^−1^ adsorption capacity noticed	Enhanced O-moieties and N-enrich functional groups favored the contaminant elimination by electrostatic interaction	^35^
Pine sawdust	550	Activation by steam	Reduce emission of greenhouse gases	Reduce the CO_2_ and N_2_O emission	Decreased enzyme and microbial activities as well as higher surface area (397 m^2^ g^−1^)	^35^
Poplar wood	300	Ball milling	Enrofloxacin	Removal capacity noticed at 80.20%	The increased photocatalytic performance of ball milled-modified-biochar was owing to the generated radicals	^37^
Orange peel waste	950	Microwave activation	Congo red	136 mg g^−1^ sorption capacity noticed	Surface functionality improved	^32^
Hickory, bagasse, and bamboo	600	Clay-biochar composites	Methylene blue	Enhancement of removal capacities by around 5 times	Electrostatic attraction (with biochar) and Ion exchange (with clay)	^20^
Corn straws	600	MnOx-doped biochar	Cu	Highest removal capacity; maximal removal capacity as high about 160 mg g^−1^	Formation of the inner-sphere complexes with MnOx and oxygen-comprising groups	^9^
Mg-accumulated tomato tissues	600	Mg-loaded biochar	Phosphate	Around 88% removal of Phosphate from the solution	Nano-scale Mg(OH)_2_ and MgO particles as core sorption sites for aqueous	^38^
Mg-enriched tomato leaves	600	Mg-doped biochar	Phosphorus	Highest removal capacity > 100 mg g^−1^	Precipitation of Phosphorus by chemical reaction with Mg-particles and surface deposition of Phosphorus on Mg-crystals on biochar surfaces	^38^
Peanut hull, hickory chips, sugarcane bagasse, and bamboo	600	Chitosan-loaded biochars	Cd, Cu, and Pb	Increased elimination of metals	Electrostatic interaction	^39^
Corn	300,450,600	Mg-modified biochar	Phosphorus	Highest removal noticed	–	^11^
Sugar beet	300	Mg-modified biochar	Phosphorus	Highest removal volume > 100 mg g^−1^	The appearance of the nano-sized MgO-particles on the biochar surfaces as active sorption sites for aqueous P	^12^
Rice straw	200–500	Mineral loaded composite by [Ca(H_2_PO_4_)_2_]), CaCO_3_, and kaolin	Carbon retention	Three minerals, particularly [Ca(H_2_PO_4_)_2_]) were effective in enhancing C retention and strengthening biochar stabilization	Increased C retention and stability of biochar with mineral loading due to increased formation of aromatic Carbon	^18^
Pinewood	600	MnO-loaded adsorbent	Pb, As(V)	Removal capacities of As(V) enhanced by around 4 and 5 times, while those of Pb enhanced by around 2 and 20 times	The occurrence of birnessite particles exhibited strong interactions with metals	^27^
Soybean straw, peanut straw, and rice straw	750	Aluminum-treated	As(V)	Al-treated adsorbents sorbed 445–667 mmol kg^−1^ at 5 pH, in contrast to slight removal on un-treated biochars	Inner sphere complexes with Al(OH)_3_ on the surfaces of treated adsorbents	^12^
Hickory chips	600	Fe-doped biochar	Arsenic	Highest removal capacity of About 2 mg g^−1^ in contrast to negligible removal on raw biochar	Chemisorption mechanism on Fe-loaded biochar	^20^
Rice hull	350	Composite with nZVI	Trichloroethylene	The degradation efficiency of Trichloroethylene was around 99% due to the nZVI-biochar composite	Higher SSA and O-enrich functional groups of nZVI-treated biochar increased SO_4_ generation and induced Trichloroethylene degradation	^6^
Rice husk	300	Fe and Ca-treated biochar	Chromium and As(V)	Observed more than 90% removal	Electrostatic interactions and heavy metal precipitation	^2^
Cotton stalk	350	Fe_2_O_3_-loading	Phosphate	Enhanced phosphate removal capacity from 0 to 0.963 mg g^−1^	Desegregation of porous trait of biochar, maximum removal ability of Fe_2_O_3_, and exceptional flow features of granular particles	^39^
Orange peel	250–700	Fe^2+^/Fe^3+^ prepared magnetic biochar	p-nitrotoluene and Naphthalene	The removal rate was higher than un-treated biochar	–	^40^
Pinewood	600	Magnetic biochar	As (V)	Higher sorption of As(V) from aqueous	γ-Fe_2_O_3_ particles on the treated adsorbent surface functioned as sorption sites by electrostatic interactions	^8^
Rice hull	400	Zinc sulfide loading	Pb	Notably increased removal capacity	–	^20^
Oak Bar, Oakwood	400, 450	Magnetic composite	Pb and Cd	Removal capacities were higher than fresh and other un-treated adsorbents	Electrostatic interactions	^30^
Cottonwood	600	Fe_2_O_3_-modified	Arsenic	The highest removal capacity of the 3147 mg kg^−1^ was noticed	Nano-colloidal structures of strong dispersed γ-Fe_2_O_3_ particles on both surface and interior of the treated adsorbent matrix	^6^
Corn straw	500	Na_2_S-modifed	Atrazine, Hg(II)	After modification, the sorption capacities for Atrazine, Hg(II) comprehensively increased	The sulfur content was markedly enhanced by 101.29% under Na_2_S treatment	^13^
Thalia dealbata	500	MgCl_2_-loaded	Cd and sulfamethoxazole	The addition of treated biochar enhanced the removal of sulfamethoxazole (by 50–58%) and Cadmium (by 24–25%) as compared with pristine biochar	SA of MgCl_2_ loaded biochar (110.6 m^2^ g^−1^) was greater than un-modified biochar (7.1 m^2^ g^−1^)	^1^
Bamboo	700	FeSO_4_, Chitosan and Fe_2_(SO_4_)_3_	Cr (VI)	127 mg g^−1^ sorption capacity was observed by modified biochar	Electrostatic attraction, reduction, chelation, and complexation	^19^
Maize straw	600	N-loading	Cd^2+^	197 mg g^−1^ adsorption capacity observed was higher than untreated biochar	Hydroxyl groups, complexation with graphitic N	^41^
*Ficus microcarpa*	500	Chitosan	Sb^3+^	167 mg g^−1^ adsorption capacity observed	H–bonding, π–π interaction, surface complexation, chelation, and electrostatic interaction	^42^
Rapeseed straw	600	MnSO_4_	Sb(V)	0.70 mg g^−1^ adsorption capacity noticed was greater than untreated biochar	Electrostatic interaction, hydroxyl/carboxyl Sb inner-sphere complexation, Sb-O-Mn complex, and physical adsorption	^43^
Populus	600	FeCl_3_	As(V)	99% adsorption efficacy was found higher than unmodified biochar	Electrostatic interaction and Fe-As precipitation	^44^
Glucose	800	N-loading	Cr(VI)	400 mg g^−1^ adsorption capacity noticed	Reduction, complexation, and physisorption	^24^
Corn straw	800	S-loading	Fe^2+^	50 mg g^−1^	Co-precipitation, ion exchange, and chemical complexation	^37^
Biological modifications						
Peanut shell	500	*hibiscicola* strain L1	Cu^2+^	45.8% removal capacity	Reduction and precipitation	^29^
Peanut shell	500	*Pseudomonas*	Cr(VI)	38.2% removal capacity, which was higher than un-treated biochar	Ion-exchange and complexation	^21^
Peanut shell	500	*Pseudomonas*	Ni^2+^	81% removal capacity was noticed, which was higher than un-treated biochar	Reduction and precipitation	^9^
Corn straw	300	*Vibrio*	Diesel oil	94%	Physical adsorption and biodegradation	^24^
Erding	500	*Bacillus cereus* LZ01	Chlortetracycline	82%	Biochar adsorption and biodegradation via LZ01	^45^

**Table 2:** Properties of modified biochars obtained from various treatments.

Modification	Biochar	Pyrolysis temperature (°C)	Experimental condition	pH	C %	H %	O%	N %	Ash %	Pore volume (cm^3^ g^−1^)	SSA m^2^ g^−1^	References
Steam activation	Tea waste	300	Un-modified	7.90	70.10	5.20	19.60	5	5.70	0.006	2.30	^10^
			Modified	8.60	71.50	4.80	18.20	5.50	6.40	0.004	1.50	
Steam activated	Invasive plant	300	Un-modified	10.90	66	5.60	23.10	5.10	25.40	0.004	0.90	^11^
			Modified	11.10	68.10	5.10	21.40	5.10	28.70	0.003	1.20	
Steam activation	Tea waste	700	Un-modified	11	85.10	2	8.90	3.9	10.90	0.022	342.2	^12^
			Modified	10.50	82.40	2.1	11.60	3.9	16.70	0.109	576.1	
Steam activation	Invasive plant	700	Un-modified	12.30	69.40	1.30	24.40	4.60	43.70	0.008	2.30	^13^
			Modified	11.70	50.60	1.70	44.90	2.50	70.70	0.038	7.10	
Zn-loading	Pine cone	500	Un-modified	–	67.90	3.90	22.10	0.5	2.1	0.016	6.60	^15^
			Modified	–	71.20	3	20.40	0.5	2.1	0.028	11.50	
FeCl_3_-modified	Wheat straw	450	Un-modified	7	47.20	2.40	18.40	1.10	–	0.012	9.50	^16^
			Modified	8.30	25.90	1.70	21.60	0.60	–	0.038	50	
Amino-modified	Sawdust	500	Un-modified	4	68.70	3.80	–	0.30	–	0.005	2.60	^17^
			Modified	6	62.10	4.20	–	4.60	–	0.005	2.50	
Methanol-treatment	Rice husk	450	Un-modified	–	70.60	3.50	24.10	0.80		1	51.90	^18^
			Modified	–	71.10	3.60	23.40	0.80		0.90	66	
KOH modification	Rice husk	450–500	Un-modified	7	42.10	2.20	0.50	12.10	42.20	0.028	34.40	^19^
			Modified	7	76.40	3.30	0.90	16.90	3.50	0.073	117.80	
H_2_O_2_ oxidation	Peanut hull	300	Un-modified	6.2	56.30	5.60	36.60	0.90	–	–	1.30	^13^
			Modified	4.4	48.30	5.80	43.80	0.80	–	–	96.90	
Clay-loaded composite (montmorillonite)	Hickory chips	600	Un-modified	–	81.80	2.20	14	0.70	–	–	401	^20^
			Modified	–	80.90	2.20	15.10	0.30	–	–	376.10	
Clay-loaded composite (montmorillonite)	Bagasse	600	Un-modified	–	76.40	2.90	18.30	0.80	–	–	388.30	^21^
			Modified	–	75.30	2.20	18.90	0.70	–	–	407	
Clay-loaded composite (Kaolinite)	Bamboo	600	Un-modified	–	80.90	2.30	14.90	0.10	–	–	375.50	^22^
			Modified	–	83.30	2.40	12.40	0.20	–	–	408.10	
CO_2_-ammonia treatment	Cotton stalk	600	Un-modified	–	–	–	–	1.10	–	0.070	224	^23^
			Modified	–	–	–	–	1	–	0.130	351	
MnOx-loading	Corn straw	600	Un-modified	–	85.30	1.70	5.20	0.80	5	0.036	61	^24^
			Modified	–	73	0.30	10.90	0.70	13.10	0.006	3.20	
Metal coating	Pulverized sub-bituminous	600–1000	Un-modified	6.4	81.60	–	17.90	–	–	0.079	190	^25^
			Modified	–	65.50	–	13.20	–	–	0.126	245	
MnO_2_-loading	Pinewood	700	Un-modified	4.5	–	–	–	–	–	0.200	369	^29^
			Modified	–	–	–	–	–	–	0.194	361	
MnO_2_-loading	Pinewood	600	Un-modified	–	85.70	2.10	11.20	0.30	4.0	0.003	209.60	^26^
			Modified	–	61.50	1.90	27.60	0.20	33.40	0.066	67.40	
CO_2_/NH_2_-modified	Cotton stalks	600	Un-modified	–	–	–	–	1.10	–	0.070	224.10	^27^
			Modified	–	–	–	–	3.50	–	0.130	351.50	
Magnetic biochar (Zero-valent iron)	Paper waste	700	Un-modified	–	–	–	–	–	–	0.083	67	^28^
			Modified	–	–	–	–	–	–	0.079	102.20	
Magnetic biochar (Fe^2+^/Fe^3+^)	Orange peel	700	Un-modified	–	67	1.50	–	2	14.90	0.390	501	^30^
			Modified	–	0.40	0.20	–	0.20	95.70	0.033	19.40	
Magnetic biochar (Fe^2+^/Fe^3+^)	Orange peel	400	Un-modified	–	65.70	3.50	–	1.80	6.90	0.041	28.10	^30^
			Modified	–	29.40	2.20	–	0.50	35	0.042	23.40	
Magnetic biochar (Fe^2+^/Fe^3+^)	Orange peel	250	Un-modified	–	56.50	5.10	–	1.70	3.10	0.059	51.60	^30^
			Modified	–	35.10	3.60	–	1.10	42.40	0.052	41.20	
Zn(NO_3_)_2_-modified	Pine cones	500	Un-modified	4.70	67.90	3.90	22.10	0.50	2.10	0.016	6.60	^31^
			Modified	4	71.20	3	20.40	0.50	2.10	0.028	11.50	
Mg-Ca loaded	Corn cob	300	Un-modified	–	35.50	6.30	–	0.70	4	–	–	^32^
			Modified	–	43.30	5	–	0.60	5	–	378	
H_2_O_2_ oxidation	Apple tree branch	550	Un-modified	9.79	72.53	14.85	2.49	1.49	–	–	6.67	^33^
			Modified	5.93	62.69	21.58	2.86	1.42	–	–	7.95	
HNO_3_-modified	Tea waste	300	Un-modified	7.16	57.80	4.42	34.12	3.66	6.15	–	–	^34^
			Modified	2.40	57.03	4.47	32.99	5.51	2.80	–	–	
Chemically modified (H_2_SO_4_)	Tea waste	300	Un-modified	7.16	57.80	4.42	34.12	3.66	6.15	–	–	^35^
			Modified	3.40	60.79	4.69	30.65	3.87	4	–	–	
Chemically modified (HCl)	Tea waste	300	Un-modified	7.16	57.80	4.42	34.12	3.66	6.15	–	–	^35^
			Modified	2.55	63.15	4.75	28.18	3.92	3.10	–	–	
HNO_3_-modified	Tea waste	500	Un-modified	7.04	69.66	2.96	24.82	2.55	11.40	–	–	^35^
			Modified	2.44	56.57	4.39	33.78	5.26	3.10	–	–	
HNO_3_-modified	Tea waste	700	Un-modified	10.09	71.03	2.11	23.74	3.12	9.26	–	–	^35^
			Modified	2.57	71.30	2.21	22.69	3.80	2.50	–	–	
Chemically modified (H_2_SO_4_)	Tea waste	500	Un-modified	7.04	69.66	2.96	24.82	2.55	11.40	–	–	^35^
			Modified	2.35	61.27	4.55	30.26	3.96	3.50	–	–	
Chemically modified (H_2_SO_4_)	Tea waste	700	Un-modified	10.09	71.03	2.11	23.74	3.12	9.26	–	–	^35^
			Modified	4.16	71.37	2.14	23.45	3.04	6.50	–	–	
Chemically modified (HCl)	Tea waste	500	Un-modified	7.04	69.66	2.96	24.82	2.55	11.40	–	–	^35^
			Modified	2.65	63.35	4.17	28.07	3.88	3.80	–	–	
Chemically modified (HCl)	Tea waste	700	Un-modified	10.09	71.03	2.11	23.74	3.12	9.26	–	–	^35^
			Modified	2.49	74.02	2.22	20.62	3.15	7.83	–	–	
HCl-modified	Peanut shells	500	Un-modified	9.20	–	–	–	–	32.54		–	^36^
			Modified	8.60	–	–	–	–	8.52	–	–	
H_3_PO_4_-modified	Swine manure	700	Un-modified	–	31.96	0.66	4.77	1.60	60.73	0.07	227.56	^37^
			Modified	–	48.35	0.66	4.41	2.23	43.98	0.09	319.04	
H_3_PO_4_-modified	Rice straw	700	Un-modified	–	31.77	0.98	7.23	0.96	58.97	0.23	369.26	^39^
			Modified	–	37.77	0.43	5.31	1.05	55.27	0.23	372.21	
H_2_O_2_ oxidation	*Eucalyptus saligna*	500	Un-modified	5.60	82.10	0.97	9.20	0.17	–	0.138	333.72	^40^
			Modified	6.48	78.40	1.29	12.70	0.14	–	0.146	347.46	
HNO_3_ + H_2_SO_4_	wheat straw	450	Un-modified	8	66.15	2.09	7.21	0.87	–	–	–	^41^
			Modified	7.30	60.57	1.60	14.82	1.21	–	–	–	
H_2_O_2_ oxidation	Yak manure	350	Un-modified	–	37.44	5.87	26.76	2.71	27.22	–	1.03	^42^
			Modified	–	40.04	3.99	29.17	2.90	23.91	–	6.36	
H_2_O_2_ oxidation	Eucalyptus wood	550	Un-modified	–	75.5	2.90	21.50	0.1	–	–	249	^43^
			Modified	–	64.8	2.30	32.60	0.3	–	–	261	
H_2_O_2_ oxidation	Corn stover	600	Un-modified	8.30	41.60	1.50	8.10	0.40	–	–	178	^44^
			Modified	7	40.60	1.30	8.30	0.40	–	–	179	
HNO_3_-modified	Pinewood chips	300	Un-modified	6.20	54.80	4.10	41	0.12	0.15	–	–	^45^
			Modified	5.80	56.50	1.20	40.30	1.99	0.11	–	–	
H_2_SO_4_/HNO_3_ treatment	Alternanthera philoxeroides	350	Un-modified	9.56	47.70	4.19	25.83	2.68	19.36	–	4.78	^46^
			Modified	2.12	60.34	1.91	28	1.85	5.46	–	7.14	

### Modification/treatment by chemicals

Chemical treatment involves both one-step and two-step modification methods. Activation and carbonization phases are attained simultaneously during one-step chemical modification in the existence of a modifying material. The two-step chemical modification involves raw feedstock’s carbonization tailed through the treatment of pyrolyzed product via mixing with modifying material e.g., various chemicals.

#### Chemical modification with acids and alkali

**Figure 3:**
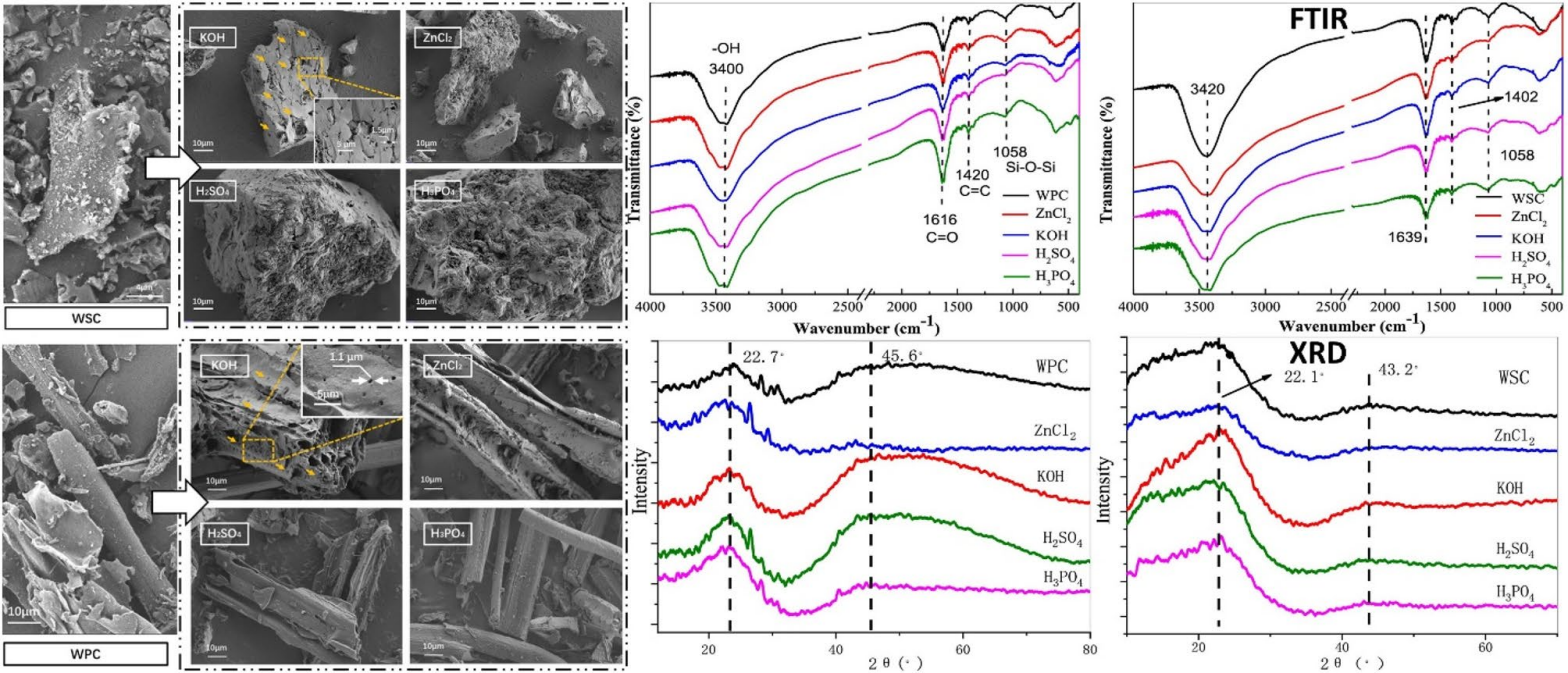
SEM images, FTIR, and XRD spectra of walnut shell biochar (WSC) and wood powder biochar (WPC) and modified ones with different acids and alkalis (After Liu et al.^14^).

The purposes of acid and alkali modification are to introduce acidic binding sites (carbonylic lactonic, phenolic functional groups) and develop a better porous structure for contaminant removal^12^. Biochar surface properties have been improved by employing chemical treatment^13^. Numerous studies have discussed the influence of acid modification on functional groups, pore volumes, and specific surface area (SSA). Soaking with strong acids such as HCl, HNO_3_, H_3_PO_4_, and H_2_SO_4_ has been examined for modification, which can increase the adsorbent surface acidities and improve the porous structure of biochar (Fig. 3).

After treatment with sulfuric acid, hydrochloric acid, citric acid, oxalic acid, or phosphoric acid, the resultant adsorbent generally possessed much higher surface area, pore volume, more hydrophobic and acidic groups for contaminant sorption^15–20^. The mechanism involves the improvement in the pore structure and specific area of the adsorbent, which has a significant effect on the physical sorption of pollutants^21^. Functional groups e.g. -COOH formed through acidic treatment also show a significant effect in the contaminant-sorption mechanism, therefore altering the removal ability of treated adsorbent^22^.

Acid treatment modifies the physicochemical attributes of biochar to increase the adsorption capacities for the elimination of inorganic and organic pollutants from soil and wastewater. The pickling mechanism decreased the sludge-based adsorbent’s micro-pore volume and enhanced the mesoporous volume, thereby improving the sorption capacity of biochar for antibiotics and heavy metals^23^. Compared to fresh biochar, H_3_PO_4_-treated eucalyptus-derived biochar exhibited higher removal efficiency of chromium hexavalent^24^. Citric acid-modified biochar showed the maximum sorption capacity of 12,109.4 and 2475 mg kg^−1^ for Pb and Cd in the soil, this capacity was greater than un-treated biochar^25^. H_3_PO_4_ is a frequently used modifying agent for acid treatment and a more eco-friendly material than other hazardous and corrosive reagents e.g., zinc chloride^26^. H_3_PO_4_ can decompose aromatic materials, aliphatic and lignocellulosic while creating polyphosphate and phosphate cross bridges to elude the shrinkage in the porosity enhancement mechanism^27^. More mineral acids including HCl, H_2_SO_4,_ and HNO_3_ have also been extensively used for biochar activation. The modification with nitric acid has been exposed to cause micropore wall degradation owing to its corrosive property, subsequently in a reduction of the surface area^28^. Comparably, H_2_SO_4_ modification caused a reduction in porosity from 12 to 40% and enhanced the distribution size of heterogeneous micropores in biochar. Organic acids e.g., oxalic acid increase the sorption of contaminants through proton-promoted and ligand mechanisms^29^. However pre-treatment with 10% H_2_SO_4_ affected the O and C contents and a mixture application of 30% oxalic acid and H_2_SO_4_ showed a 250-fold enhancement of surface area than untreated biochar^30^. Similarly, the HCl-modified biochar of wheat straw showed more heterogeneous pores compared to un-treated biochar^31^. Indigenous metal/inorganic contaminants can also be efficiently eliminated by acid application^32^. Generally, it is documented that modification with acids can establish the various functional groups having acidic contents e.g. amine and carboxyl groups, thus increasing the metals removal capacity and affinity by surface complexation and cation exchange with these more active sites.

Dai et al.^29^ applied H_2_SO_4_-modified bur cucumber adsorbent for sulfamethazine removal in soil. A high water–solid partition coefficient of 229 L kg^−1^ was noticed for loamy soil. Both chemisorption onto hemiacetal functional groups and chemical diffusion into pores were supposed to be retention processes. Table 3 sums up the chemical oxidation and acid/alkali modification approaches for biochars described.

**Table 3:** Chemical oxidation and base/acid modification process of the biochar.

Biochar Feedstock	Modification	Ratio of Liquid–solid	Temperature (°C)	Concentration	pH	Time	References
Bamboo	NaOH	–	60	10%	–	6 h	^29^
hydrothermal biochar, Alamo switch grass	KOH	500 mL: 2 g	25	2 M	5 and 7	1 h	^26^
Rice hull	KOH	500 mL: 2 g	25	3 M	–	1 h	^27^
Rice husk	H_2_SO_4_	200 mL:20 g	60	10% (v/v)	5 and 9	1 h	^28^
Burcucumber	C_2_H_2_O_4_ + H_2_SO_4_	100 mL: 5 g	25	30%	–	4 h	^30^
Jarrah, Sawdust	KOH	40 mL g^−1^	90	0.1 M	–	1 h	^31^
Acacia saligna	H_3_PO_4_	40 mL g^−1^	90	1 M	–	1 h	^32^
Peanut hull	Oxidation (H_2_O_2_)	20 mL:3 g	25	10%	–	2 h	^33^
Bamboo	Oxidation (H_2_O_2_)	1 mL g^−1^	25	15–30%	–	12 h	^34^
Apple tree branches	Oxidation (H_2_O_2_)	1:20 (w: v)	80	15%	–	6 h	^35^
Tea waste	H_2_SO_4_	100 mL:10 g	60	10%	7	1 h	^36^
Tea waste	HNO_3_	10 mL: 10 g	60	69%	7	3 h	^37^
Tea waste	HCl	100 mL:10 g	50	5 M	–	24 h	^38^
Poultry manure	H_3_PO_4_	5.63 mL: 240 g	25	50%	–	2 h	^39^
Bamboo hardwood	NaOH	100 mL:10 g	40	0.40 M	–	16 h	^40^
Cow manure and wheat straw	HNO_3_	300 mL:10 g	90	25%		4 h	^41^
Swine manure and rice straw	H_3_PO_4_	40 mL:20 g	25	14%	–	24 h	^42^
Dairy manure	NaOH	5:1	65	2 M	–	12 h	^43^
Coconut shell	HCl	250 mL:5 g	20	1 M	–	3 h	^44^
Corn straw	Na_2_S	500 mL:2 g	80	2 M	–	4 h	^45^
Corn straw	KOH	500 mL:2 g	80	2 M	–	4 h	^46^
Thalia dealbata	MgCl_2_	100 mL:10 g	25	1 M		0.5 h	^30^
Auricularia auricular dreg	Cetyl trimethyl ammonium bromide (CTAB)	250 mL:5 g	25	3.0%		2 h	^40^

The main objectives of alkaline modification are to improve the amount of O-comprising functional groups such as ether, carbonyl, carboxyl, and hydroxyl as well as the specific surface area of raw biochar, therefore enhancing the removal of several contaminants^11^. Alkaline activation is a mechanism including the basic (alkaline-nature) solution applied to change biochar structure at pre -or-post carbonization stages^33^. The most commonly used alkaline agents are sodium hydroxide (NaOH) and potassium hydroxide (KOH)^34,35^. Alkali activation of biochar using NaOH and KOH can enhance the surface basicity and O content while dissolving condensed organic matter (such as cellulose, and lignin) and ash to aid subsequent modification^36^. After alkaline activation, blocked pores are cleansed, causing higher porosity^24^. Biochars with very large SA have been observed after being functionalized by NaOH and KOH^37^. Potassium species such as K_2_CO_3_ and K_2_O may be generated during modification due to the intercalation of potassium ions in the crystallite layer that creates condensed carbon structures. K_2_CO_3_ and K_2_O may diffuse into the internal structure of the adsorbent matrix expand available pores and form new different pores of product^38^.

**Figure 4:**
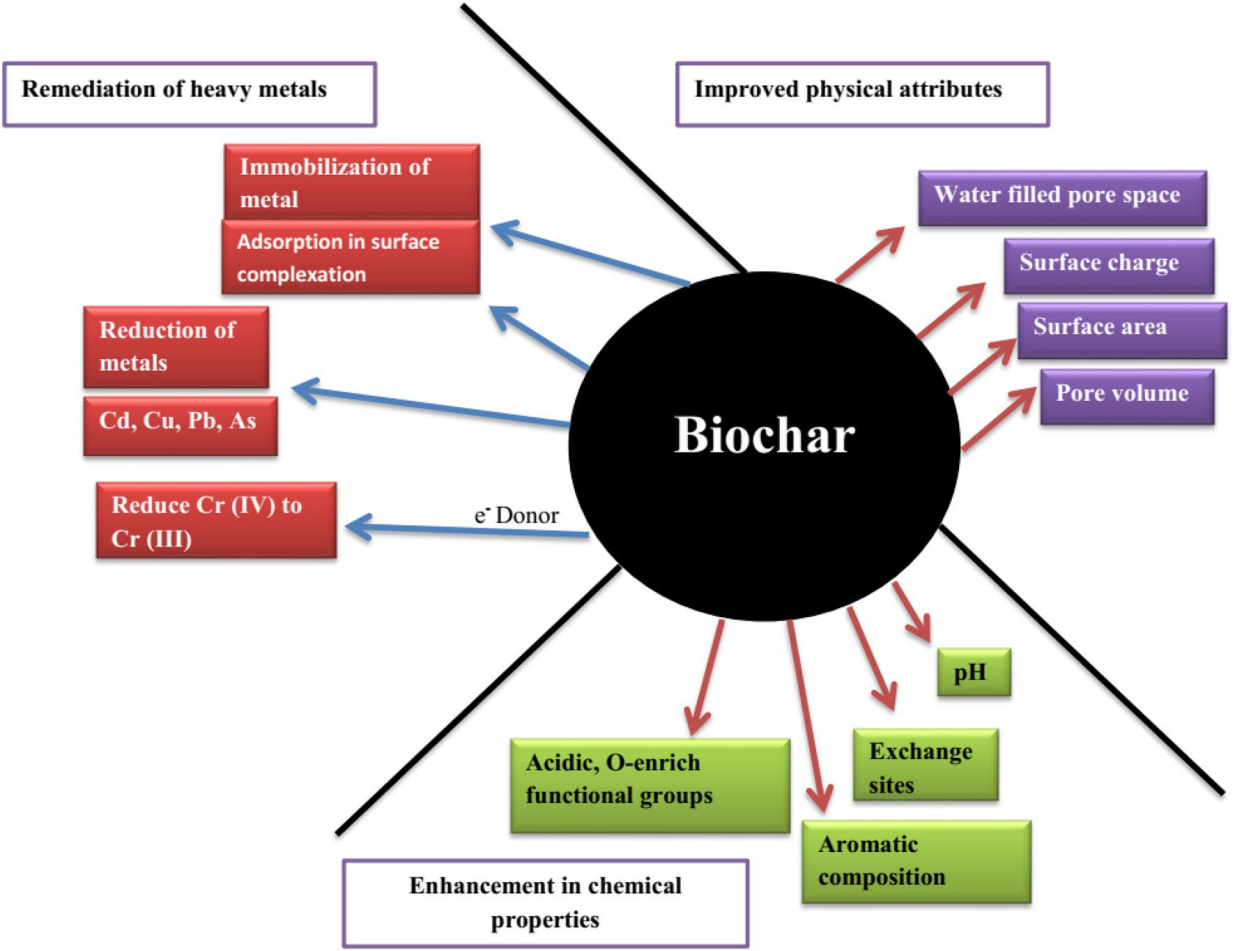
Improved performance of multiple-modified biochars.

Chemical activation of adsorbent may increase its pollutant sorption capability by forming abundant and additional sorption-sites on improved SA, providing biochar surface more conducive to surface precipitation, surface-complexation, and electrostatic attraction, and providing higher affinity for sorption and strong sorption capacity via sturdy interactions with ether, carbonyl, carboxyl, and hydroxyl functional groups^39^. Figure 4 and Table 4 encapsulate the mechanism and improved ability of the chemically activated adsorbent for contaminants sorption. However, most surface attributes prevailed unaltered after the KOH-soaking, Cd^2+^, and Cu^2+^ sorption was notably increased which may be owing to a large number of oxygen-containing functional groups and higher surface area^40^. Moreover, amination and carboxyl groups modification of adsorbent also greatly increased Cu^2+^ adsorption by powerful surface complexation with NH_2_-functional groups, which were greatly selective and slightly affected via competing cation^32^. KOH-treated biochar significantly increased its adsorption capacity to As (V) due to the enhancement of pore volume and surface area and the alternation of surface functional groups^23^.

**Table 4:** The mechanisms and enhanced performance of chemically oxidized and base/acid modified biochars.

Modification	Pollutant	Sorption capacity	Improvement	Functionalities	Mechanism	Reference
NaOH	Chloramphenicol	Around 2100 mg kg^−1^	Enhance surface graphitic C and oxygen-enrich groups	Carboxyl and carbonyl groups	Formation of H-bonds among N-comprising groups in adsorbent surface and chloramphenicol, Electron-donor–acceptor π-π interaction	^22^
KOH	As (v)	30.98 mg g^−1^	More than about 1.3 times higher removal capacity	Carbonyl group	Ion exchange	^21^
KOH	Cd and Cu	34 and 31 mg g^−1^	Approximately 20 times higher removal capacity than other raw biochars	Aromatic carbon	Surface precipitation, ion exchange electrostatic attraction, and surface complexation	^23^
H_2_SO_4_	Tetracycline	23.26 mg g^−1^	Larger SSA and porosity, higher oxygen and carbon contents, lower ash content	O-containing functional groups	Π-π electron donor acceptor interaction between the treated adsorbent surface and aromatic ring	^25^
H_2_SO_4_ + oxalic acids	Sulfamethazine	183–229 L kg^−1^	–	–	Ligand- and proton-enhanced processes; Increase in SA by acid modification	^29^
H_3_PO_4_	–	–	Enhance water extractable organic carbon content	–	Hydrolysis of ester groups formed during the pyrolysis	^26^
(Oxidation) H_2_O_2_	Pb	22.82 mg g^−1^	Removal for Pb was 20 times greater than un-treated biochar	Carboxyl groups	Enhance carboxyl groups expedite the formation of bound complexes with Pb	^27^
(Oxidation) H_2_O_2_	Hg	1470.5–1347.9 µg g^−1^	Substantially higher removal capacity than other un-treated biochars (380–618 ng mg^−1^)	Ester, Carboxyl, and Carbonyl groups	Higher SSA and total pore volume Surface O-enrich functional groups increase electron transfer, and Hg-oxidation and allow chemisorption centers	^28^
Acetic acid + Na_2_S_2_O_4_ + H_2_SO_4_	Cu	15.97 mg g^−1^	Enhancing removal capacity in fixed-bed and batch experiments	Amino groups	Ion exchange	^40^
HNO_3_ + H_2_SO_4_	Cu	12.47 mg g^−1^	Around 4.62% N was present on the treated adsorbent, fivefold enhancement of removal capacity	Amino groups	Foundation of outer-spherecomplexes with amino groups of treated biochar	^41^

Elimination of organic contaminants could be increased through π-π EDA interaction between aromatic rings of pollutants and biochar^13^. Another study reported the removal capacity of tetracycline (58 mg g^−1^) via KOH-treated adsorbent was markedly higher than reported in other studies (5 to 54 mg g^−1^), while the noticed capacity for the Chloromycetin elimination via NaOH-treated char was remarkably greater than un-activated adsorbent^22,24,30,41^. Conversely, urea treatment could produce N-enrich functional groups as well as enhance the basic nature of the surface, therefore increasing π-π dispersion forces for carbolic acid sorption^27^. He et al.^42^ presented that oxalic and sulfuric acid-modified biochar delivered a better result of 183 to 229 L kg^−1^ sulphadimethyl pyrimidine removal in various kinds of soil, maybe due to enhancement of surface areas and surface functional groups in activated adsorbent (Table 1).

#### Modification by an oxidant (chemical oxidation)

Oxidant treatment can enhance the content of O-containing functional groups, stimulating the complexation of heavy metals such as Cd, Zn, Cu, Pb, etc.^43^. H_2_O_2_ activation of manure biochar enhanced the carboxyl contents (101%) and oxygen contents (63%) of treated biochar, while the content of ash was reduced by 42% after modification. The oxidant-modified biochar could remove Zn, Cu, Cd, and Pb efficiently, which was due to the shifting of the adsorption process from precipitation to complexation. Nonetheless, H_2_O_2_ treatment was inefficient in methylene blue sorption^44^. After hydrogen peroxide activation of pinewood chip biochar, the sorption capacity of methylene blue decreased as the O-rich groups weakened the forces of delocalized π-interaction which was the core process for methylene blue sorption^38^. Apart from potassium permanganate which has a direct impact in enhancing cation-π interaction and O-containing functional groups in modified biochar^43,44^, hydrogen peroxide could also be applied as an activation agent^45^. The effectiveness of this technique depends mainly on the target pollutant type and pollutant elimination process. It is hypothesized that this technique is appropriate for metal stabilization in the soil as there is an increased surface complexation due to enhancement in O-comprising functional groups.

The hydrophilicity and functional groups of adsorbent can be activated with chemicals to fit the explicit necessities of environmental safety including the elimination of pollutants from soil and water^46^. In general, biochar derived at low temperatures has more C–H and C=C functional groups^27^. Chemical treatment using H_3_PO_4_, NHO_3_, H_2_O_2,_ and KMnO_4_ and a mixture of H_2_SO_4_/HNO_3_ can generate acidic-content functional groups (e.g., phenolic, lactonic, carbonyl, and carboxyl) on C surface at comparatively low temperatures^47^. A substantial amount of oxygen-enrich functional groups were formed via chemical activation using nitric acid compared to potassium permanganate, showing a resilient oxidizing ability of nitric acid^32^. In addition, H_2_O_2_ treatment was able to enhance the carboxylic group from 2 to 8% by oxidizing the carbonized structure of the adsorbent^22^.

N-rich functional groups (such as pyridinic, pyrrolic, lactam, imide, and amide) and oxygen-comprising functionalities play a significant role in the environmental implication for their potent complexation attractions, particularly for the base metal cations e.g. Cd, Zn, Cu^48^. Formation of the N-bearing functionalities could be achieved through nitration followed by dwindling on the carbon surface^20^. Nitro-groups are consequently decreased to amino groups on the surface by applying Na_2_O_4_S_2_ (reducing agent). Surface amination leads to the formation of amino-based groups, which stimulate basic attributes and strong attractions to impurities^28^. Applying chitosan as a modifying agent, established amine functionalities on the surface of biochar to enhance its adsorption capacity and affinity to inorganic pollutants^49^. Chitosan loading on the surface of biochar can also enhance its effectiveness as a soil rectification, as well as chitosan-loaded adsorbent, may be applied as an efficient, eco-friendly, and low-cost adsorbent to decontaminate the pollutants from the environment^50^. H_2_O_2_-modified biochar derived from peanut hull surface enhanced oxygen-enrich functional groups, particularly the carboxyl group which accelerates the metals removal capacity and affinity of adsorbent^9^. Another study found that amino-treated char with enhanced functional groups e.g., C–N, N–H, C–O, and CH_2_ could efficiently eliminate copper from wastewater because copper was intensely complexed with amino-functionalities on the surface of adsorbent^18^. Besides, char activated through KOH enhanced oxygen-comprising functionalities such as COOH, C=O, C–O, and O–H and consequently increased the tetracycline adsorption capacity^40^. At pH 7, oxygen-rich functional groups on the alkali-treated biochar accelerated the formation of hydrogen bonding with tetracycline molecules thus increasing its removal capacity^41^. Modification of biochar with hydrogen peroxide has been depicted to contain a great amount of oxygen-comprising functional groups and efficiently eliminate heavy metals such as Cd, Ni, Cu, and Pb^26^.

### Organic modification/activation by organic solvents

**Figure 5:**
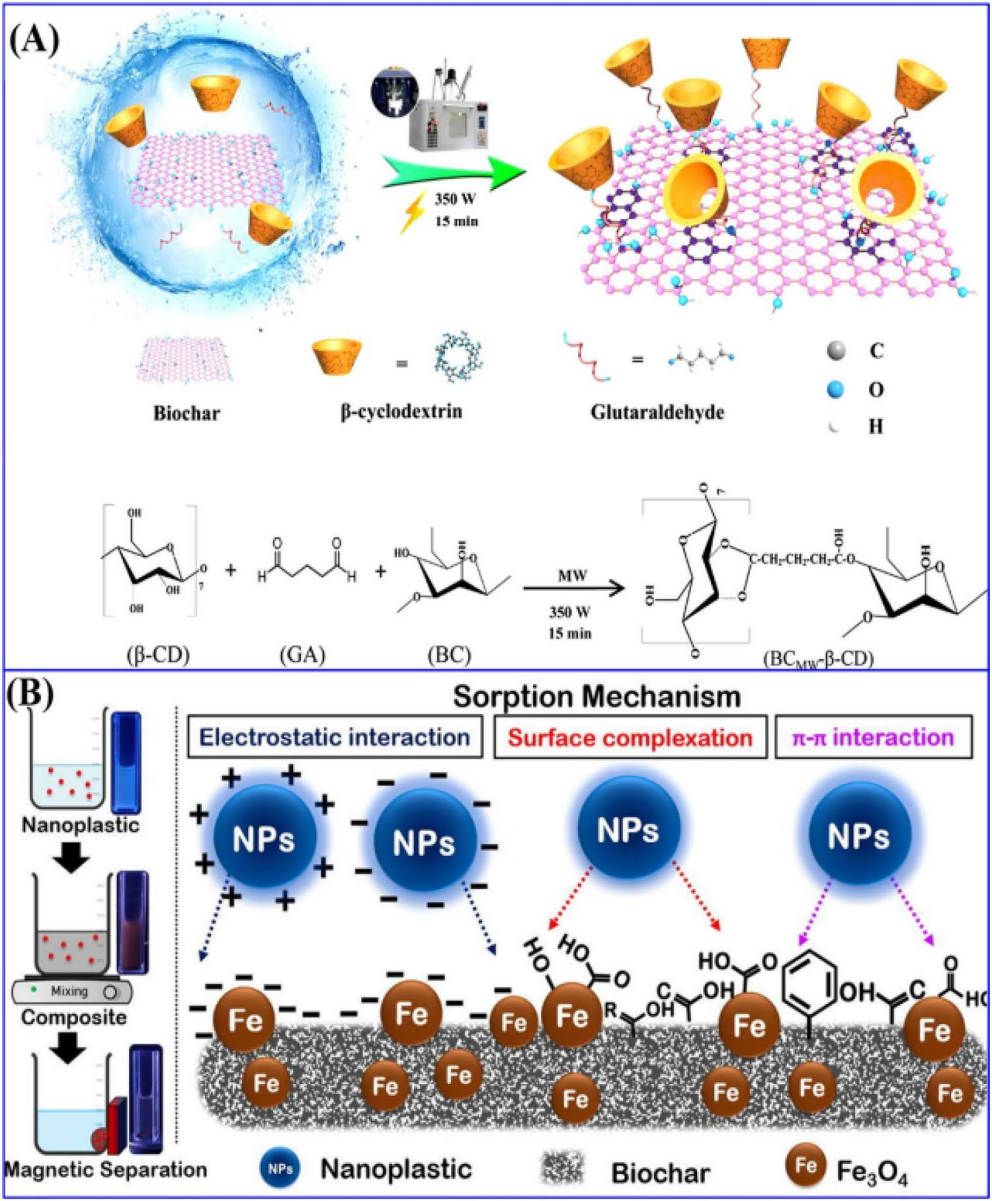
(**A**) Schematic sketch and chemical reactions of microwave-assisted one-pot synthesis of β-cyclodextrin composite biochar^19^. (**B**) The interaction mechanism between magnetic biochar and nanoplastics^15^.

It is conceivable to promote the types and quality of functional groups in biochars by mixing biochars with an organic substance containing a large number of functional groups^27^. The sorption capability also can be improved by elevating the number of sorption sites^51^. Out of various substances, chitosan has been applied in many studies. Chitosan is a natural polysaccharide that is produced from the Crustacea shell and is rich in –O,-OH, -NH_2_ functionalities. The removal capability of contaminants can be raised via doping chitosan onto the char’s surface^51^. Boraah et al.^52^ examined how the sole utilization of textile residue biochar coated with chitosan influenced cadmium-contaminated soil on cadmium distribution in horseradish trees. Plant-available cadmium in the soil showed the best efficiency in reducing cadmium concentration in the soil by 58% than the control sample. Braghiroli et al.^53^ stated that the composite of biochar and chitosan showed a great removal ability of ammonium, with a maximum range of 149.25 mg g^−1^, comparatively higher than raw biochar. Moreover, reported that the amount of surface functional groups can be improved via activated chars with macromolecules e.g. cyclodextrin, Polyaziridine, lignin, and humic acid, these provide better elimination capacity for impurities^52^. Figure 5 displays how β-cyclodextrin coated rice biochar was produced appropriately by employing a microwave-based one-pot method. The coated composite was used for concurrently removing Pb (II) and bisphenol-A^53^. Microwave irradiation could ascertain surface alteration in 15 min and the generated biochar microwave-assisted-β-cyclodextrin composite (BCMW-β-CD) showed higher sorption efficiency with a theoretical monolayer uptake of 240 mg g^−1^ for lead and a heterogeneous removal volume of 209 mg g^−1^ for bisphenol A in the monocomponent system. Carboxyl-group activation also can be achieved by applying water-soluble methanediimine and esterification by methanol^11^. The application of methanol for carboxyl alteration is low-cost. For instance, biochar derived from rice soaked with sodium hydroxide and consequently treated with methanol exhibited an improvement in surface attributes. Chemical mechanisms involved in char activation by methanol are esterification and then direct reaction between biochar carbonylic functional properties with methanol^38^. Methanol treated-adsorbent was enriched in hydroxyl and ester groups than un-activated char, which contributes to the EDA interaction formation between adsorbent surface and pollutants (pharmaceutic manufacturing wastewater, contaminated water, and soil)^54^. Jin et al.^55^ presented that methanol-treated adsorbent was more efficient in TC elimination compared to un-treated char owing to functional group modification as well as enhancement of oxygen-enrich functionalities on the adsorbent. The process proposed by the researchers was the EDA interaction generation between hydroxyl groups or ionized moiety of activated char and electron-depleted sites.

### Modification with surfactant

Surfactants are categorized into Gemini, non-ionic, anionic, and cationic surfactants rendering to a hydrophilic nature. Generally, surfactants are employed as additives in washing detergents and industrial fabrication in environmental cleaning, as well as recently extensively used as chemical agents to alter the surface attributes of several solid substances e.g. zeolite, bentonite^56^. Owing to the surface negatively charged of biochar, cationic-surfactant can smoothly capture through biochar via exchange with ample exchangeable cations (K^+^, Na^+^, and Mg^2+^), and electrostatic attraction in the char-matrix, and consequently, a surfactant-char composite/complex is generated^57^. For instance, the adsorption of cationic surfactant 1-hexadecyl pyridinium chloride on granular charcoal was mostly by ion exchange at a lower level. The partial monomolecular layer may be established with an enhancement of the cetylpyridinium chloride amount in the solution. Further, it enhanced cetylpyridinium chloride level, hydrophobic interaction among hydrophobic chains of cetylpyridinium chloride, and char improved sorption of cetyl pyridinium chloride^58^. Kumar et al.^59^ described the influences of cationic surfactants on pentachlorophenol adsorption by biochar and activated carbon. Cationic-surfactant cetrimonium bromide was merely mixed in a solution comprising pentachlorophenol, and the char-activation mechanism through CTAB occurred thru ion exchange concurrently with adsorption of pentachlorophenol on biochar-CTAB composite. Therefore, cationic-surfactants can be applied as an efficient material to alter char to improve the elimination of the anionic contaminants. Non-ionic surfactants also can be removed through charcoal by physisorption mechanism as showed through low free energy alterations in adsorption. Around 300 mg g^−1^ of Triton X-100 was doped on the charcoal^60^. Labanya et al.^61^ also observed a fixed degree of adsorption of non-ionic surfactant Triton X-100 onto the adsorbent. Contrastingly, owing to electrostatic repulsion, both micellar and mono-molecular anionic surfactants are not fluently sorbed on the biochar surface. For instance, the fragile removal of anionic surfactants on charcoal was noticed^62^. However, substantial removal of anionic surfactant sodium dodecyl sulfate was recorded on modified charcoal^48^. Labanya et al.^61^ observed that after CTAB treatment of biochar, pentachlorophenol removal capacity of activated adsorbent reduced with enhancing aqueous CTAB concentrations. This may be because of the hindrance of the hydrophobic sorption site via CTAB sorption. Contrastingly, solubilization and mobilization of pentachlorophenol by CTAB in the solution might contribute to the reduction of pentachlorophenol adsorption onto the adsorbent. The existence of cationic-surfactant CTAB greatly reduced the adsorption capacity of thiodiphenylamine onto modified char. Apart from, the sorption-site-hindrance mechanism; competition of cationic surfactant with cationic thioridazine hydrochloride interdicted the adsorption of thioridazine hydrochloride on char. Nonetheless, the occurrence of anionic surfactant only marginally reduced thioridazine hydrochloride sorption, while non-ionic surfactant Triton X-100 improved thioridazine hydrochloride sorption^22^.

### Doping of biochars

Recently, doping of biochars by metal-oxides has been applied to enhance the characteristics of biochars and consequently improve their removal performance. Doped biochar can be obtained by biochar mixed with clays, carbonaceous materials (carbon nanotubes, graphene oxide), and metal oxides, to change the surface traits of biochar. Biochar doping is distinguished from chemical modification because it involves the formation of totally new surface functional groups that were not previously present on biochar surfaces.

#### Doping with metal oxides

The aim of this method to produce doped biochar with metal oxides is to confirm a homogenous spread of metal over the surface of biochar. The biochar is used as a porous carbon support upon which metal oxides precipitate to enhance the surface area of the metal oxide. In general, doping with metal oxides of biochar is performed via soaking biochars in solutions of metal chloride and nitrate. The most used doping agents in literature are MgCl_2_, Fe (NO_3_)_3_, Fe, and FeCl_3_^63–65^. After mixing biochar with metal oxides or salt solutions, the mixture is heated under oxygenated conditions at temperatures 100–400 °C to allow chlorides or nitrates to be driven off as Cl_2_ and NO_2_ gases and alter the metal ions to metal oxides^9^. Fu et al.^66^ prepared Fe- and cobalt-soaked bamboo biochar for the elimination of the metal from wastewater. Charcoal of bamboo was mixed in a 100 mL solution comprising iron (III) chloride, cobalt nitrate, and 9 M nitric acid, followed via carbonized by microwave at 640 W for some minutes. Moreover, Mg(OH)_2_-loaded wheat-straw adsorbent was produced by applying NaOH and MgCl_2_ solutions^63^. The iron-impregnated char had markedly enhanced hydroxyl-functional groups than un-coated char due to the creation of Fe oxides on the adsorbent surface^64^. Chen et al.^65^ reported that soaking municipal waste and rice husk adsorbents in FeCl_3_, iron powder, and Cao before pyrolysis generated Fe^3+^, Fe^0^, and Ca-coated biochars. These modifications improved the removal capacity of biochar for Cr (IV) and As(V) from wastewater. Most metal oxide loading results in a decrease in the surface area of biochar owing to pores blockage by metal oxide precipitates^63^. Bamboo biochar-coated with tetrabutylammonium bromide Fe_3_O_4_, FeCl_3_ and used for the removal of polybrominated diphenyl ethers. The findings showed that coated biochar was more efficient for polybrominated diphenyl ether removal compared to other uncoated biochar^67^. Besides, Fe^3+^-loaded char was synthesized using ferric chloride salt. Doping adsorbent/char by Fe^3+^ significantly improved As(III) and As(V) removal capacities^68^. The removal capacity of Magnesium oxide-loaded biochar for the anionic dye was greatly enhanced compared to untreated biochar by around 5 times. This was due to the surface being positive in the solution after MgO-loading, which increased the removal of anionic dye. Moreover, a large number of functional groups were observed on coated biochar, which aided the elimination of dye^66^. Thakur et al.^51^ described a substantial enhancement of Cr(III)-oxyanion sorption via cobalt-loaded bamboo biochar rather than unmodified biochar. The cobalt-loaded showed higher pore volume and surface area compared to uncoated biochar, which led to greater sorption capability. Nonetheless, microwave heating and nitric acid were also involved during the preparation process of cobalt-loaded composite^66^. Coated biochar was synthesized with the oxides of Mg, Mn, and Al and observed increasing sorption of metal cations (Pb) and oxyanions (P and As) and metal cations (Pb)^58^. Fu et al.^66^ prepared a biochar with great removal capacity for P and As from wastewater via modification with aluminum chloride to form an adsorbent-AlCOOH composite. Hemavathy et al.^43^ modified the biochar with MnCl_2_ and noticed that the treated biochar showed significantly increased adsorption capacity for Pb and As compared to pristine biochar.

#### Non-metallic heteroatom doping (emerging technique)

Premarathna et al.^69^ reported that the non-metallic heteroatom loading is an advanced approach to modifying char by influencing its electronic characteristics, therefore increasing its catalytic sorption ability for pollutant remediation. Previous studies involving non-metallic materials-loaded-biochar and related environmental implications and mechanisms are detailed in Table 5. As reported, the most frequently employed non-metallic elements for modification of biochar include iodine, phosphorus, boron, sulfur, and nitrogen, and can be used to eliminate the aqueous pollutants^70^. According to Chen et al.^71^ nitrogen is extensively used heteroatom in char modification. The nitrogen-doping technique can increase the electrochemical functioning of char by creating amine-N, pyridinic-N, and pyrrolic-N, graphitic-N species during the modification mechanism^72–75^. Cheng et al.^72^ reported that graphitic-N could stimulate the transfer of electrons within the C skeleton, therefore increasing the catalytic capability of Nitrogen-loaded char for the persulfate-activation. Petrovic et al.^73^ presented that the pyridinic-N and pyrrolic-N bonds can perform as electron donors and concurrently create N-defects which render more active sites in nitrogen-loaded char. Qiu et al.^74^ demonstrated that the amine-N could work as a binding site for metals-ions through chelation results. Thus, nitrogen-doped/loaded biochars have been used as efficient catalysts in the elimination of heavy metals, dyes, oxybenzene, and antibiotics in the aquatic system (Table 5). For example, Cheng et al.^72^ produced nitrogen-doped biochar and it exhibited high sorption capacity for methyl orange (906 mg g^−1^) and methylene blue (326 mg g^−1^). Theoretical calculations and spectroscopic studies verified that introduced pyridine-N and pyrrole-N had significant effects on dyes elimination and suggested mechanisms e.g., H-bonding, electrostatic attractions as well as π-π stacking^72^. In addition, N-doped/loaded biochar exhibited high potential in the catalytic capability for contaminant removal^72–75^. For instance, Deng et al.^75^ described that the N-doped biochar exposed high N content (13%) and large surface area (738.50 m^2^ g^−1^), and exhibited a strong catalytic capacity for per-oxymoron-sulfate activation to eliminate cotrimoxazole, with an elimination rate of 90% in a half hour. As observed through electron paramagnetic resonance spectra and quenching tests, ^1^O_2_ was noticed to be the leading reactive species favoring sulfamethoxazole degradation and non-radical oxidation involving electron transfer and ^1^O_2_ was the leading removal mechanism^76^. Rangabhashiyam et al.^77^ reported that Sulfur doping provides a modified adsorbent with additional functional groups including C–S–O, C=S, and C–S–C, C–S as well as sulfur rings. In particular, sulfur-containing groups can reinforce the spin density of surrounding C atoms, consequently enhancing the catalytic performance of the char^43^. Moreover, the C–S–O structure in char can prompt the nucleophilic addition of per-oxymoron-sulfate to create copious ^1^O_2_^78^. After S-doping, modified biochar has exposed a great affinity towards inorganic elements. For instance, Rangabhashiyam et al.^77^ manufactured a hierarchical Fe-maize straw biochar composite, and then Mn^2+^ and S^2−^ were concurrently introduced, creating a ternary Fe–Mg–sulfur-biochar composite. This composite was examined for Pb^2+^ removal from an aqueous solution, and they concluded that the Sulfur-doping increased the removal of Pb^2+^ through PbS precipitation. Dinh et al.^79^ reported that the Sulfur-loading could introduce Phenoxytamol radicals (C–O**·**) and vacancy defects on the acacia-derived adsorbent, which expedited peroxymonosulfate activation to degrade 90% of bisphenol A in half an hour. Raman analyses and scavenging experiments confirmed that Sulfur-loaded char prompted the creation of surface-bound **·**OH and ^1^O_2_ which led to the efficient elimination of BPA Diphenylolpropane^79^.

**Table 5:** Removal of contaminants and associated mechanisms through non-metallic heteroatom-doped biochar.

Doping agent	Biochar type	Pyrolysis temperature (°C)	Contaminants	Removal capacity	Mechanism involved	References
Nitrogen	Bamboo	500	Chlorotetracyclin	92%	Non-radical pathways ^1^O_2_ Radical pathway: SO_4_**·**^−^	^68^
Boron	Wheat straw	700	Phenol	33 mg g^−1^	Non-radical pathways ^1^O_2_ Radical pathway: SO_4_**·**^−^	^70^
S and N	Peanut shell	300	Diethyl phthalate	14 mg g^-1^	Increased removal via pyridinic-N formation and the oxidized sulfur groups on doped-biochar	^71^
Nitrogen	Pomelo peel	800	Sulfamethoxazole	95%	Non-radical oxidation involving electron transfer and ^1^O_2_	^72^
Sulfur	Tapioca peel	800	Rhodamine B Malachite green	33 mg g^−1^ 30 mg g^-1^	H- bonding, surface interaction, and electrostatic attraction	^73^
Sulfur	Wood pellets	800	Bisphenol A	91%	Driven via hydroxyl radicals and surface-bound singlet O_2_	^71^
Co-doped (boron and nitrogen)	Wheat straw	700	Oxytetracycline	60%	High defect sites and large SSA	^75^
Sulfur	Bamboo	600	Oxytetracycline	89%	Non-radical pathways ^1^O_2_ Radical pathway: SO_4_**·**^-^	^77^
Boron	Wheat straw	900	Sulfamethoxazole	90%	Boron-doping restrained the electron transfer	^78^
Co-doped (copper and nitrogen)	Glucose	700	Tetracycline	100%	Radical degradation such as electron transfer and **·**OH	^84^
Nitrogen	Hickory chip	600	Reactive red	37 mg g^-1^	zeta potential enhancement and electrostatic interaction	^79^
Co-doped (nitrogen and sulfur)	Wood shaving	800	Methylene blue	40%	Activation through the thiophenic S and graphitic-N active sites	^74^
Nitrogen	*Enteromorpha prolifera*	800	Phenanthrene Acenaphthene Naphthalene	90 mg g^−1^ 51 mg g^−1^ 86 mg g^−1^	Partition effect, π–π stacking, mass transfer, and pore-filling	^81^
Nitrogen	Glucose	700	Pnitrophenol	94%	New sorption sites of pyrrolic-N and pyridinic-N	^75^
Nitrogen	Maize straw	600	Methyl blue Acid orange 7	436 mg g^−1^ 292 mg g^−1^	π–π stacking and pore-filling Lewis acid–base interaction, π–π stacking, and electrostatic attraction	^33^
Nitrogen	*Phragmites australis*	280	Phenanthrene	1.9 mg g^−1^	Electrostatic attraction, hydrophobic effect, and π–π interaction	^82^
Nitrogen	Alfalfa	600	Methyl orange Methyl blue	326 mg g^−1^ 906 mg g^−1^	H-bonding, electrostatic interactions, and π–π stacking	^69^
Nitrogen	Sawdust	800	Bisphenol A	50 mg g^-1^	π-π EDA interactions	^67^
Nitrogen	Pomelo peel	200	Orange II	100%	^1^O_2_ and **·**OH expedited the degradation	^72^
Nitrogen	Peanut shell	350	Pb^2+^	130 mg g^−1^	ion exchange and surface complexation	^23^
Co-doped (phosphorus and nitrogen)	Lotus leaf	600	Pb^2+^	321 mg g^−1^	Precipitation and surface complexation	^32^
Sulfur	Corn straw	800	Pb^2+^	181 mg g^−1^	Precipitation, reduction, and complexation	^69^
Nitrogen	Loofah sponge	400	Cr (IV)	238 mg g^−1^	In-situ reduction, complexation, and electrostatic attraction	^44^
Nitrogen	Hemicelluloses	200	Cr (VI)	349 mg g^−1^	Chelation, redox, and electrostatic attraction	^71^
Nitrogen	Maize straw	600	Cd^2+^ Cu^2+^	197 mg g^−1^ 104 mg g^−1^	Complexation and cation-π bonding with hydroxyl groups and graphitic-N	^74^
Boron	Maize straw	800	Fe^2+^	50–132 mg g^−1^	Co-precipitation, ions exchange, and chemical complexation	^75^
Co-doped (nitrogen and oxygen)	Rice husk	500	Zn^2+^ Ni^2+^ Cu^2+^	12 mg g^−1^ 8 mg g^−1^ 13 mg g^−1^	Electrostatic attraction and surface complexation	^80^

Boron (B) is another excellent material heteroatom that can restrain electron dispersion modify the surface characteristics of biochar and offer additional defect sites^80^. Doping of maize straw-biochar with boron enhanced the O_2_-enrich functional groups and SSA (890 m^2^ g^−1^), which eventually enhanced its capability to adsorb Fe^2+^^81^. The contact mechanisms involved between boron-treated biochar and Fe^2+^ were co-precipitation, ion exchange, and chemical complexation. Respecting the catalytic influence of boron-treated biochar, Murtaza et al.^82^ applied the boron-doped biochar derived from wheat to activate peroxy-disulfate for sulfamethoxazole elimination and degradation of sulfamethoxazole significantly (up to 90%) was found in 120 min. Theoretical and experimental results explained that the introduced boron species could perform as Lewis acid sites to increase the surface affinity towards peroxydisulfate and that the char-facilitated electron transfer process was mainly accountable for the non-radical route^82^. In another study, B-doped biochar (B-BC) was prepared using boric acid. The modified biochar had more porosity and SSA up to 897.97 m^2^ g^−1^. Among the modified biochars prepared, the maximum adsorption of Fe^2+^ (132.78 mg g^−1^) was noted at 55 °C using 800B-BC_1:2_ due to chemisorption, co-precipitation, and ion exchange^18^. A spontaneous endothermic physical adsorption process was recorded in the case of neonicotinoid adsorption while using boron-doped porous biochar prepared through hydrothermal carbonization. Electrostatic and hydrophobic interactions were noted between acetamiprid and porous biochar used with a maximum adsorption capacity of 227.8 mg g^−1^ for acetamiprid^83^.

Moreover, several studies reported that co-doping of two materials on the char. Copper and nitrogen co-doped char synthesized and was applied for tetracycline degradation, and composite biochar showed better performance compared to raw biochar, Cu-doped, and N-doped biochar^72^. In another study, Singh et al.^49^ prepared three kinds of doped biochar (co-doping of S and N, S-doped and N-doped) biochar produced from bamboo. Conversely, they observed that the degradation amount of antibiotics via sulfur and nitrogen-co-doped biochar (70%) was less than sulfur-doped biochar (89%) and nitrogen-doped (90%). The main cause for this mechanism was that nitrogen-doped biochar had the maximum amount of **·**OH and SO4**·** − , higher defects, and higher SSA^32^. Although substantial advances in non-metal component-loaded biochars have been attained, it is still in infancy with great ability for melioration in environmental decontamination. The non-radical process behind persulfate activation is still unclear, which demands more research. Moreover, complex sample fabrication, poor reusability, and the high cost of this kind of biochar require to be accurately addressed in future exploration.

### Modification by carbonaceous nanomaterial

#### Coating with carbon nanotubes

Biochar in combination with carbon nanotubes, comprising functional groups can generate resilient bonds with contaminants and biochar surface^85^. Han et al.^86^ reported that the carbon nanotube shows significant physicochemical characteristics such as greater π-π interactions, large surface area, magnificent thermal conductivity, superior electron mobility, and higher mechanical strength. These properties are helpful for the sorption of several contaminants and work as a perfect catalytic supplement for the removal of impurities. Thus, carbon nanotube shows a substantial potential to be applied in the processes of remediation. Jiang et al.^87^ produced a composite of carbon nanotube with sludge biochar to remove sulfamethoxazole. Compared with pristine biochar, the composite exhibited a higher surface area (119 m^2^ g^−1^), and maximum sorption capacity. The study of physicochemical characteristics, kinetics, thermodynamics, isotherms, and various environmental factors showed that its remarkable removal efficiency was mainly ascribable to pore filling, π-π conjugation as well as the interaction of functional groups^87^. Chen et al.^58^ presented the methylene blue adsorption capacities of carbon nanotube doped biochar and untreated bagasse and hickory biochar. The maximal sorption capacities of nano-tube biochar composite and untreated biochar (5.5 and 2.4 and mg g^−1^ respectively) were about twice the time higher than untreated biochar. Methylene blue took up greater affinity binding sites within the carbon nanotube. Moreover, the char exhibited the capability to eliminate Phenothiazin-5-ium via itself, when sorption-sites of carbon nanotube were fulfilled. The electrostatic interaction was the leading process for Phenothiazin-5-ium sorption and microporous diffusion governed its sorption amount^30^. Jiang et al.^88^ observed that the SA of carbon nanotube-doped biochar derived from Giant cane was very low compared to un-doped biochar, but a large amount of acidic-functional groups were noticed on the surface of carbon nanotube-doped adsorbent. These acidic functional groups may smoothly interact with lead and generate a firm type to immobilize it on a coated-biochar surface, which improves the adsorption capacity of lead. Jin et al.^55^ observed that the cobalt-doped biochar of bamboo showed higher pore volume and surface area than the un-doped adsorbent, which led to greater chromium hexavalent removal capacity. Similarly, a substantial increase in pore volume and surface area was also found with an enhancement of Fe quantity on coated biochar^50^. Loading of surfactant for carbon nanotube dispersion during the preparation of carbon nanotube doped-biochar resulted in a greatly higher sorption capacity of carbon nanotube-loaded biochar for pollutants (lead and sulfapyridine) than without surfactant owing to the magnificent distribution and dispersion of carbon nanotube on the adsorbent surface^11^. Captivatingly, no noticeable competition was found between sulfapyridine and lead, signifying the site-specific sorption of both pollutants on carbon nanotube-doped adsorbent surface^15^. Further, pollutant sorption and hydrogen storage capability were assessed on multi-walled carbon nanotubes that were loaded on bamboo biochar using microwave plasma to improve chemical vapor deposition. Nonetheless, only little enhancement was noticed compared with un-doped biochar because of the lower hydrogen storage ability of multi-walled carbon nanotubes than pristine biochar^89^. However, carbon nanotubes are very efficient for pollutant elimination due to their nanostructure and large surface area, high cost, and inconvenience for engineering applications limiting their use. Thus, biochar could aid as a mesoporous/microporous carrier of carbon nanotubes to develop new recyclable and effective sorbents for wastewater and polluted soil treatment.

#### Using graphene for modification

Graphene modification has attracted both engineers and scientists after its discovery for its special two-dimensional structure and novel traits, such as electrical and thermal conductivity, surface area, and mechanical strength^90^. Compared to carbon nanotubes, difficult recovery and separation for reuse limit the extensive application of graphene in wastewater and polluted soil remediation. To overcome these drawbacks, graphene-based composite covering particles are produced and biochar is one of the promising materials as a carrier of graphene. Production of graphene-doped adsorbent also typically follows the two-step dip-coating process as above, e.g. peanut shell-derived feedstock was soaked in graphene solution to absorb graphene and then pyrolyzed by slow pyrolysis in an N_2_ environment^74^. Ghanim et al.^91^ observed the sorption enhancement of methylene blue and phenol via graphene-doped biochar. Higher pore volume (0.55 cm^3^ g^−1^) and surface area (11.30 m^2^ g^−1^) after coating graphene on cotton-biochar may be the major reasons for enhanced sorption. Moreover, π-π bonding between methylene blue or phenol graphene sheets contributed to enhancing sorption capacity. Hafeez et al.^92^ found a substantial enhancement of methylene blue on graphene soaked-biochar (almost 20 times greater), and strong π-π bonding between methylene blue and graphene on biochar surface was believed to be the leading mechanism for the improvement of methylene blue sorption via graphene-doped biochar. Graphene loading on biochar introduces a large amount of oxygen-enrich functional groups such as carboxyl, hydroxyl, and carbonyl creating the binding between biochar surface graphene. For the recover and regeneration of graphene adsorbents, simple desorption processes were sufficient using ethanol and deionized water as eluents. However, the regenerative properties of graphene-based adsorbents have not been rigorously investigated and are required to be explored in future studies for the sustainable circular economy.

### Physical modification

Generally, mechanical/physical modification techniques are usually economically feasible and simple but are less efficient compared to chemical modification. The physical modification method uses various oxidizing agents e.g., CO_2_, air, and stream. Physical modification has been considered an effective method to improve biochar functionality by influencing hydrophobicity, polarity, and surface functional groups of biochar^44^. Nonetheless, the drawbacks of physical activation techniques include a long time (~ 4 h) for activation and more energy consumption (10.6 to 58.0 kcal)^89^. For these drawbacks, chemical activation is considered the primary option for biochar engineering. Future studies should focus on filling the research gap to address the drawbacks of physical activation.

#### Activation by steam

Activation by steam is a common modification process used to improve the structural porosity of biochar and eliminate contaminations e.g. products of incomplete combustion. This method helps to enhance the surface area upon which sorption can proceed. Activated poultry manure biochar was produced at 700 °C followed by steam modification at 800 °C with a range of water-flow rates and durations. Greater flow rates and longer treatment times enhanced the sorption of Zn, Cu, and Cd on the surface of the adsorbent^93^. However, activation with steam increases the porosity and surface area of the biochar, found in a study conducted by Ghassemi-Golezani and Rahimzadeh^94^ the copper removal capacity of slow pyrolysis-derived biochar of Miscanthus was not notably changed by modification with steam at 800 °C. They noticed that while steam activation of treated biochar enhanced the surface area, many functional groups were reduced, alongside a rise in aromaticity. Likewise, Ghazimahalleh et al.^95^ noticed that sawdust-derived biochar activated by steam activation enhanced the surface area but had a slight effect on the properties of functional groups. The steam activation did not affect the removal capability for phosphate owing to electrostatic repulsion via the negatively charged surface of the adsorbent. Thus, concerning inorganic contaminant removal, activation with steam looks to be more efficient when employed before a second modification step that forms functional groups, as the steam only improves the surface area of the adsorbent.

#### Gas purging

Biochar modification with high-temperature CO_2_-ammonia mixture application has been examined to adsorb gases (CO_2_) obtained NH_3_ and CO_2_ treated-biochar under a range of creation temperatures^96^. After the manufacture of biochar from the cotton stalk, it was slowly heated up to a specific temperature (500–900 °C) in a quartz reactor with N_2_ purging and then NH_3_ or CO_2_ was purged. The ammonification could introduce Nitrogen-comprising groups onto biochar and enhance Nitrogen content up to about 3.90 wt% in CO_2_-ammonia treated biochar, although CO_2_ application could play a substantial role in the pore formation and amend the micro-porous structure of adsorbent accelerating gas sorption capabilities of the biochar^23^. Krerkkaiwan et al.^96^ presented that the pore volume and surface area of the CO_2_-treated biochar were higher than the untreated biochar. CO_2_ could react with the carbon of biochar to form CO, thereby forming microporous structures. At ambient temperature, the gas sorption capability of CO_2_-treated biochar was markedly higher than that of untreated biochar^97^. The removal capacity of treated biochar exhibited a linear relationship with micro-pore volume and the process of CO_2_ sorption was recognized as physical sorption^18^.

#### Ball milling

Ball milling is a low-cost and eco-friendly technique that has been used for biochar modification through the improvement of key attributes such as pore structure, specific surface area, and enhancement of various surface functional groups than to the un-modified biochar^38^. Previously published literature has reported that several operational parameters could influence the physicochemical and catalytic/adsorptive characteristics of resultant biochar such as media mass ratio: biochar, dry or wet milling (without or with solvent), reaction time, and solvent characteristics, milling temperature and speed, reaction atmosphere and ball size distribution^98^. Furthermore, the grinding media shape (cubes, ellipsoids, and spherical balls) may affect the milling mechanism but spherical balls are the most reliable grinding media^99^. Recently, remediation of pollutants from the environment has been examined by ball-milled modified biochars. For example, Haider et al.^100^ reported that the ball milling technique expedited the mixing of Al/Mg layered double hydroxide into the matrix of biochar, and ball-milled Al/Mg layered double hydroxide-biochar composite exhibited a higher adsorption capability for Cd^2+^ (119 mg g^−1^). Kasera et al.^89^ reported that the ball-milled modified-biochar has also shown prominent potential for the remediation of organic pollutants such as phenols, dyes, and antibiotics, and other inorganic pollutants including phosphate and ammonia as well as heavy metals and metalloids. The main mechanisms behind this modification included pore structure, specific surface area, and enhancement of various surface functional groups. In addition, emerging applications employing ball-milled modified- biochars have focused on soil remediation and thermal/photocatalysis^13^. However, the ball-milling approach is a new research field to synthesize modified biochar; it is still in its beginning. Thus, further research is required to determine critical research directions and control or reduce the existing challenges.

#### Microwave activation/modification

Pokharel et al.^68^ reported that Microwave modification is an advanced technique based on high-frequency electromagnetic radiation with frequencies (from 0.03 to 300 GHz). Microwave irradiation creates dipole rotation at an atomic scale, therefore producing heat energy within the constituents/materials^47^. This activation technique allows both outer and inner biochar surfaces to be heated concurrently without direct interaction at low temperatures and results in the production of microwave-modified biochar with a larger surface area and various functional groups than raw or inactivated biochar^101^. Microwave modification resulted in rough and lamellar morphology of the modified biochar, enhanced fraction of micropore volume from 9.01% at 400 °C to 60.25% at 900 °C, and BET surface area from 19 to 1722 m^2^ g^−1^. The modified biochar had somewhat irregular mesopores (2.3–11.9 nm), and abundant functional groups such as β-CD, − OH, and –COOH. Ma et al.^102^ stated that microwave-modified biochar was more efficient in reducing the phytotoxicity of PAHs and heavy metals in peppergrass compared to pristine biochar. Moreover, microwave modification for biochar also accelerated the removal of Congo red and methylene blue from wastewater. As an instance, Maaoui et al.^103^ described that the combined application of steam and microwave activation created highly effective modified biochar with a greater surface area (570 m^2^ g^−1^), and resulted in an efficient performance in the methylene blue removal with a maximum sorption volume of 38.5 mg g^−1^. Nevertheless, the environmental utilization of microwave-modified biochar is still scanty, which is mostly ascribed to the expensive equipment operation and maintenance^103^.

### Impregnation with clay mineral and mineral oxides

As a novel idea, modified biochar has been produced by biochar doped with minerals^104^. Various minerals can accelerate biochar efficiency, among which clay minerals such as vermiculite, montmorillonite, and attapulgite have attracted great attention (Table 6) because of their great ion exchange capability and large pore structures for different contaminants under aquatic ecosystem^105^. Clay minerals have been widely used for pollutant elimination due to their composition, mineralogical structure, surface charge, and cation exchange capacity. The clay-loaded biochar obtained from a biomass mixture with montmorillonite and kaolinite showed lower surface areas than untreated biochar, this was because of pores blockage in biochar through clay minerals^106^. Kaolinite, gibbsite, and montmorillonite are the most frequently used clay minerals, as a cost-effective adsorbent^107^. Lv et al.^108^ carried out a study on, the biochar of hickory chips, bagasse, and bamboo doped with clay particles (kaolin and montmorillonite) to improve its functionality. They observed the mineral-doped biochar composite showed a higher porous structure after modification. Lyu et al.^109^ observed that the surface complexation between hydroxyl groups and Pb (II) provided via montmorillonite stimulated the sorption efficiency. Sonowal et al.^50^ produced porous magnesium oxide-doped biochar with thick flakes of polycrystalline magnesium oxide from several feedstock biomasses such as peanut shells, sugar beet, cottonwood, sugarcane bagasse, and pinewoods. These biomasses were dipped in magnesium dichloride solutions and ensued dry-mixture of magnesium dichloride incorporated biomass was heated at 10 °C min^−1^ at 600 °C. The use of N_2_ treatment is necessary to eliminate by-product gases such as hydrochloric acid and thus accelerate the generation of magnesium oxide particles in the biochar matrix. Additionally, the direct application of Ca/Mg-contained tomato tissues is another novel method of creating Mg-comprised biochar, which enriches Mg(OH)_2_-particles and nano-sized magnesium oxide within the biochar matrix^110^. Besides, Magnesium oxide and birnessite doped biochars derived from pine were produced via two modification ways to enhance sorption capacity for Pb(II) and As (III)^111^. Pre-dripping pine wood feedstock in Mn(II) Chloride solution and resulting pyrolysis yielded magnesium oxide doped biochar, although birnessite doped biochar was prepared by soaking of pine-wood char with birnessite by precipitation adopting pyrolysis process^112^. The potassium permanganate mixing with biochar markedly changed the pore volumes and surface area of the biochar. A significant reduction in surface area was found, whereas the width of pores enhanced from 23 to 92 nm with enhancing potassium permanganate (KMnO_4_) doping^113^. These structural alterations of modified biochar may be because of the demolition of nano-pore structures and deformation from nano-pores into macropores/mesopores by potassium permanganate, which typically performs as an impregnable oxidizing agent^113^. Murtaza et al.^114^ observed the enhancement of oxygen-enrich functional groups in magnesium oxide-doped biochar. In addition, most of the surface oxygen in magnesium oxide doped-biochar was bonded to magnesium (Mn) in the form of Mn-OH and Mn–O (26.3%, and 63.9% respectively). XPS examination showed the presence of hydroxyl, Mn–O, Mn–OH, and C–OH on the surface and chemisorbed water on the surface of biochar^114^. Modification-caused alterations in the elemental composition of the biochars are detailed in Table 6. Biochar doped with kaolinite and montmorillonite significantly improved the iron and aluminum contents than un-doped biochar, although the carbon, hydrogen, nitrogen, sulfur, and oxygen contents were significantly higher than un-doped biochar^115^. On the other hand, the surface oxygen content of magnesium oxide-doped biochar was greatly higher (40%) compared to un-doped biochar (15%), showing the enrichment of oxygen-comprising functionalities^116^. MnOx-doped biochar has greater thermal stability compared to untreated biochar, owing to the transition of MgO through the heating mechanism^117^. The magnesium oxide-doped biochar showed much greater sorption capability to copper (II) (160 mg g^−1^), which was higher than the un-doped biochar (19 mg g^−1^). Generation of inner-sphere complexes with magnesium oxide and oxygen-enrich groups, cation-π- bonding, and cation exchange are the main processes involved in improving the sorption of copper (II) on the magnesium oxide-doped biochar^118^.

**Table 6:** Recent advances in biochar modification by minerals and their effectiveness in environmental application.

Objectives	Modify agent	Environmental application	Key results	References
Provide a large number of hydroxyl groups	Montmorillonite	Adsorption of atenolol (86.86 mg g^−1^) and Pb (II) (139.78 mg g^−1^)	Amino O and amino N produced hydrogen bonds on the modified biochar surface	^104^
Enhance the surface area	Montmorillonite	Immobilization of Zinc, Copper, and lead in soil	FTIR and sorption experiments results showed that chemisorption was the prevalent immobilization process	^105^
Improve the surface area	Montmorillonite	Tetracycline sorption (77.96 mg g^−1^)	The leading sorption mechanism was Physisorption	^107^
Precipitate Pb	Hydroxyapatite	Pb immobilization in the soil	The residual fraction of lead enhanced by 66% after the addition of modified biochar	^108^
Stimulate surface complexation and enhance surface area	Goethite	Immobilization of phosphorous, cadmium, roxarsone, and arsenic in the soil	Co-precipitation, surface complexation, redox reaction, and ion exchange attributed to the immobilization process	^109^
Enhancement of pore structure	Diatomite	Methylene blue sorption capacity (153 mg g^−1^)	The modified biochar has numerous pore channels in the mesoporous area, supporting the dye sorption	^110^
Produce active oxygen species via S and Fe addition	Hematite	Norfloxacin sorption (about 1.90 mg g^−1^)	Hematite loading successfully produced · SO^−^4 and –OH, stimulating the norfloxacin degradation	^111^
Increase ion exchange for anions	Mg–Al	Phosphate sorption (80.43 mg g^−1^)	the adsorption capacity of phosphate enhanced with the enhancement of the Al^3+^/Mg^2+^ ratio due to weakened inter-layer charge density and widened inter-layer space	^112^
Increase ion exchange for anions	Zn-Al	Phosphorus sorption (152 mg g^−1^)	Inter-layer complexation and anion exchange were major sorption mechanisms	^113^
Increase ion-exchange	Mg–Al	Methylene blue sorption (406 mg g^−1^)	Within 20 min the adsorption mechanism could reach an equilibrium	^114^
Promote anions for co-precipitation	Mg-Fe	Sorption of lead (476 mg g^−1^)	Co-precipitation among hydroxyl groups of surface and Pb (II) was the leading sorption mechanism	^115^
increase ion exchange	Attapulgite	Immobilization of cadmium and arsenic in sediment	Compared with pristine biochar, modified biochar had greater pore volume, surface area, higher CEC, and a large amount of O-enrich groups	^116^
Increase ion exchange	Attapulgite	Sorption of oxytetracycline (33 mg g^−1^)	Complexation, ion exchange, and hydrogen bonding contributed to great sorption capacity	^117^

### Electrochemical modification

To generate modified biomass/biochar adsorbents, an easy-to-use and promising alternative method to chemical solution-based methods is electrochemical modification. Electrochemical modification is a rapid and simple technique aimed at acquainting the specific functional groups and soaking chemicals onto the pristine biochar surface. Typically, this technique uses a two-electrode electrochemical cell where the modifying agent doesn't need to be added from an outside chemical source thanks to a sacrificial anode like Fe. The first step in the modification process is to run an electric current between the electrodes in an electrolyte solution while stirring the biomass and/or biochar components around in the solution. The anode dissolves to release modifying agent ions (Fe^2+^), which then proceed through a sequence of reactions to deposit the appropriate modifying agent (iron oxide, for example) on the biomass/biochar surface^54^.

For target pollutants like As the electrochemical modification process parameters must be optimized to provide an adsorbent with the maximum adsorption capacity. For instance, it has been noted that the density of sites on the adsorbent surface and the particle structure affect the affinity of iron oxides, such as goethite, magnetite, and hematite, for arsenate, As(V)^119^. Moreover, altering the preparation procedure's pH and temperature can alter the structure of the iron oxide particles produced during the modification process^120^. Another important factor that is directly related to the concentration of solution iron that may be deposited on the surface of biomass or biochar is the length of the applied direct current.

A simple and unique electrochemical alteration that can simultaneously improve porosity and surface functioning has been developed more recently to find alternative methods^54^. For instance, biochar produced by aluminum electrode-based electro-modification followed by pyrolysis demonstrated superior phosphate adsorption capability over other chemically modified adsorbents due to an increase in the formation of a crystalline structure on the surface with nano-sized AlOOH^115^. Although powdered biochars have a remarkable potential for phosphate adsorption, their low hydraulic conductivity makes them unsuitable for continuous fixed-bed column adsorption systems and makes them difficult to collect and separate from an aqueous solution at the post-adsorption step^121^.

Islam et al.^44^ synthesized a magnetic biochar of corn straw under an electric field produced via an electrode. The external electric field facilitates the rod-like crystalline iron oxide (Fe_3_O_4_) nano-particles to disseminate rigorously into inner pores of biochar, resulting in an enhancement of pore diameter and a slight reduction of SSA^44^. Electrochemical modification techniques can also be applied for magnesium oxide (MgO) impregnation^28^. Using graphite as the electrode and magnesium dichloride as the electrolyte, magnesium dichloride nano-particles were disseminated and enhanced on the surface of marine macroalgae derived-biochar. The resultant biochar/magnesium oxide composite was exhibited to remove phosphate efficiently, achieving a higher removal capacity of about 90%^28^. MgO-loaded biochars are efficient for the soil metal stabilization mechanisms^122^.

### Magnetic modification

Magnetic biochars have been extensively used as a sorbent for soil and wastewater remediation. After activation with ferromagnetic elements such as Ni, Fe, and Co their oxides, biochars can be recycled easily by an external magnetic field, making the cleaning and regeneration procedures much easier. Many studies have scrutinized the sorption mechanisms and re-usability of magnetic adsorbents^123^. The multi-functional attributes of biochar indicate its potential as an efficient sorbent for pollutants in soil, wastewater, and water systems, thus modified biochar has been manufactured through a magnetic loading process to improve its sorption capacity of anionic pollutants^124^. Production approaches, sorption efficiency, and reusability of the magnetic adsorbent have been studied elsewhere^72^. Due to the ability to produce reactive oxygen species including hydroxyl radical, sulfate radical, and hydrogen peroxide magnetic adsorbent can be applied for catalytic degradation of organic pollutants. For example, Li et al.^119^ produced a magnetic biochar of pine needles with Mn-Fe binary oxides and scrutinized its capability for the degradation of naphthalene in polluted water. The redox potential of Mn(III)/(II) is greater than that of Fe(III)/(II), signifying that in this Fenton system, Mn(III) could be decreased via Fe(II) efficiently, thus ceasing the limitation of hydrogen peroxide fabrication on Mn(III) reduction. Thus, the transfer of electrons in this system can be elevated, resulting in more than 80 times greater naphthalene decomposition capacity compared to untreated biochar^119^. The surface areas of magnetic modified biochar produced via chemical co-precipitation of Fe^2+^/Fe^3+^ were lower than non-magnetic biochar, although the average pore diameter of modified biochar was higher compared to un-modified biochar^125^. This is due to magnetic loaded-biochar containing a substantial amount of ferric oxide, which has abundant transitional pores and low surface areas^125^. The hybrid adsorption characteristic of this magnetic-loaded biochar accelerates the effective elimination of phosphate and organic contaminants simultaneously. The stability and presence of magnetic in magnetic char for a long period supported the possibility of magnetic separation after utilization, which is a main benefit for the wastewater remediation process^120^. Rajput et al.^126^ prepared a magnetic biochar via thermal pyrolysis of iron (III) chloride-activated biomass. The resulting modified biochar has nano-sized (< 5–60 nm) or colloidal maghemite particles embedded in a porous biochar matrix and therefore showed excellent ferromagnetic attributes with a saturation magnetization of 69.2 emu g^−1^. Additionally, sorption results exhibited that magnetic loaded-biochar has a higher sorption capability to arsenic (III) in solution. Due to its ferromagnetic characteristic, the spent/exhausted biochar can be smoothly collected and separated via magnetic separation^126^. The introduction of ferric oxides facilitated the creation of inner-sphere complexes, resulting in reduced bioavailability and mobility of the heavy metals^127^. Besides plant growth, metal stabilization can also be accelerated, but the processes involved in this mechanism remain unclear. It is proposed that magnetic loaded-biochars could also be applied for the degradation/adsorption of organic pollutants in contaminated soil due to the formation of reactive oxygen species^128^.

#### Photocatalytic modification

Photocatalytic degradation of organic pollutants could be attained by biochars after doping with metal oxide-based semiconductors e.g. Titanium dioxide, cuprous oxide, copper (II) oxide, and Zinc oxide^121^. Metal oxide doping on biochar can be accomplished in several methods, for example, sol–gel, hydrothermal, and hydrolysis^129^. Pan et al.^130^ synthesized Titanium dioxide-doped biochar using a low temperature; the resultant TiO_2_-supported biochar attained great phenol degradation efficiency^130^. Biochar could not only assist as a host substance for metal semiconductors but also accelerate the process of electron transfer. For example, the mesoporous structure of biochar derived from walnut shells confirmed the dispersion of Titanium dioxide nanoparticles^131^. Another research carried out by Premarathna et al.^69^ used TiO_2_-Zn doped biochar for the degradation of sulphamethoxazole under visible light. Due to the electro-negativity of the adsorbent, intermediates, and sulphamethoxazole come into interaction with the photo-catalyst more smoothly, and the produced electron, can then transfer to the biochar surface without the recombination of the electron–hole pairs, consequently stimulating the photocatalytic mechanism. In particular, biochar performs as an electron pursuer in the conduction band of semiconductors that can promote the separation of electron–hole pairs and the process of electrons^132^.

In comparison to the Cu2O-CuO sample, the Cu2O-CuO@BC-1.0 composite showed lower values of band gap energy for CuO (1.70 eV) and Cu_2_O (2.10 eV). Due to the high surface area and small band gap, the Cu2O-CuO@BC-1.0 composite performed better photocatalytically than its separate equivalents, BC and Cu2O-CuO. After 90 min of photocatalysis, the Cu2O-CuO@BC-1.0 composite was able to degrade RO29 with a 94.12% efficiency at the starting concentration of 20 mg/L and pH 8.9. After 90 and 180 min of treatment, 47.31% and 79.62% COD elimination efficiencies were reported, respectively^111^.

#### Iron modification

Iron modification or activation technique for biochar is projected for two main purposes: i) strengthening the separation efficacy to reuse and recycle the biochar, and (ii) increasing the decontamination capability of biochar by the interaction between the target contaminants and loaded Fe^39^. Iron-activated biochar has been synthesized using iron oxides such as goethite and hematite, nano zero-valent iron, and iron sulfide^133^. Iron-activated biochar can be created by precipitation, ball milling technique, thermal reduction, and co-pyrolysis^8^. Considering previous studies, the utilization of Iron-activated biochar can be classified into three types including a catalyst, reductive agent, and adsorption material. Hematite and goethite are the most commonly employed Fe minerals to increase the adsorption efficiency of biochar for heavy metals such as Hg, As, Cr, Cu, Pb, and Cd^134^. Lima et al.^135^ produced the goethite-modified biochar and observed maximum sorption capacities for As^3+^ and Cd^2+^ were 78 mg g^−1^ and 63 mg g^−1^, respectively. Baser et al.^40^ synthesized three types of Fe-activated biochars including Fe_3_O_4_- modified biochar, Fe_2_O_3_- activated biochar, and Fe-treated biochar via one-step ball milling of magnetite, ferric oxide, and iron powder, respectively. In the elimination trial for antibiotics, Fe_3_O_4_-activated biochar exhibited the highest sorption capacities for tetracycline 90 mg g^−1^ and carbamazepine 60 mg g^−1^. Fe-activated biochar has also exposed great efficiency in redox reactions of metals such as U(VI), Cr(VI), and As(III) and organic pollutants to reduce their toxicity^136^. Biochars modified via FeOOH, FeS, and nZVI have been examined for reductive degradation since they can provide reductive agents such as Fe (II), S (II), and Fe^0^ species. Gautam et al.^64^ observed that the nZVI-activated biochar exhibited effective sorption ability (54 mg g^−1^) for Cr(VI). The decline of toxic Cr^6+^ to less toxic Cr^3+^ was also observed, which highlighted the significant role of Fe^0^. The excellence of nZVI-activated biochar over un-activated biochar was confirmed for sulfamethazine removal because nZVI-activated biochar expedited the free radicals generation, which favored the sulfamethazine degradation^14^. Fan et al.^33^ stated that peanut hull biochar activated/engineered via starch and FeS minimized labile U(VI) to non-labile U(IV) species and the main role of S(II) and Fe^0^ in the reduction mechanism was highlighted through the XPS study. Another dynamic direction for the utilization of Fe-treated biochar is in the organic pollutant’s degradation, particularly in persulfate and Fenton-like activation systems. Oxidant activation such as ozone, permanganate, persulfate, H_2_O_2_, and via Fe-treated biochar has received great attention^137^. Due to great electron shuttling capability, abundant oxygen-enrich functional groups, and persistent free radicals (PFRs), Fe-activated biochar has been confirmed for its potential as a Fenton-like catalyst in catalyzing hydrogen peroxide to create hydroxyl radicals to remove organic pollutants in water and soil systems^138^. Moreover, PFRs, Fe^2+^ on Fe-activated biochar can act as efficient activators to create reactive oxygen species such as SO_4_^**·**−^, ^1^O_2_, and **·**OH which can efficiently degrade the several types of organic contaminants such as phthalate esters, metronidazole, bisphenol A and tetracycline^139^. For example, with the assistance of Electron spin resonance analysis, Fan et al.^34^ verified that the application of Iron-activated biochar derived from sugarcane bagasse favored the formation of reactive oxygen species in a Fenton-like system and **·**OH radical quenching trials exhibited that the elimination efficiency reduced by around 85%, indicating the leading role of **·**OH in metronidazole degradation. Furthermore, iron activation can result in a slenderer band gap of biochar, which supports the photocatalysis/adsorption for aqueous methylene blue, F^−^, Pb^2+^, and Cr^6+^^140^. Compared to other metal salts and oxides, iron is the most abundant element on the earth, more economical, and less toxic for the environment. Thus, Fe-activated biochar is suggested as an amendment in the remediation of polluted soil systems owing to such advantages. Fe-modification/activation has been the most widely practical and advanced tailoring method in the remediation of environmental pollutants by biochar at large-scale utilization.

#### Biological modification/bacterial inoculation of biochar

Compared to chemical and physical modification techniques, biological techniques for biochar modification are less researched, mainly because of their more complex functioning properties. Initially, authors employed anaerobic bacteria to transform biomass into biogas and applied the resulting digested residue for biochar synthesis^141^. Biochar produced from anaerobically digested biomass residues shows superior characteristics such as higher SSA, high pH, and resilient ion exchange capacity and has exposed greater removal efficacy for heavy metals, organic contaminants, phosphate, etc.^142^. Furthermore, a comparatively novel method employing the biological post-treatment technique to immobilize the microbes on biochar has attracted recently^143^. Biochar has been suggested as a superior carrier for microbe inoculation due to its simple preparation, economical, nutrient enrichment, and high porosity^144^. Biofilm theory proposes that living cells can release a wide range of polymers and attach themselves to the surface of biochar, creating a microbial biofilm surrounded via an extracellular matrix that accelerates the pollutant's degradation/ adsorption^145^. Dou et al.^32^ examined the elimination potential of Bacillus cereus- inoculated biochars derived from pharmaceutical wastes for chlortetracycline. The maximal elimination rate of chlortetracycline was found 85%. The microbial inactivation test exposed that the chlortetracycline removal mechanism was mainly governed via chemisorption and microbial degradation through biochar^48^. Increased bioremediation of diesel oil was described by Labanya et al.^61^, they used Vibrio-loaded biochar to clean the diesel oil-polluted seawater, and they found superior removal efficiency as compared to the control. Menzembere et al.^146^ verified that biochar-inoculated with three strains including Citrobacter sp., Bacillus cereus, and Bacillus subtilis could efficiently immobilize Cd (II) and U (VI) in the polluted soil. Relative to the control, the diethylenetriaminepentaacetic acid-extractable concentration of Cd and U in the soil reduced by 56% and 69%, respectively and bacteria-modified biochar decreased metal uptake, thus stimulating the growth of celery.

## Effectiveness of multiple-modified/engineered biochars in soil system remediation

### Inorganic contaminants (metalloids/metals)

Owing to the extensive anthropogenic actions, the agricultural/farming soils are contaminated with a wide range of inorganic contaminants such as metalloids/metals and organic contaminants^147^. Engineered/modified biochar is believed as an economical and efficient soil-remediation candidate to reduce the environmental stress of polluted soils^142^. The remediation process includes the indirect process (enhanced soil characteristics via engineered biochar) and the direct process (degradation/ immobilization of pollutants via modified biochar) in multipart terrestrial systems^148^. The various engineered/modified biochars and their remediation efficiency for metalloids/ metals in soils are documented in (Table 7). Sarkar et al.^149^ amended a cadmium-contaminated soil (20 mg kg^−1^) with 5% chitosan and fabric waste biochar combined application of fabric waste biochar and chitosan (1:1 w/w) and chitosan-coated fabric waste biochar, respectively. They observed that the chitosan-biochar application exhibited the greatest efficacy in reducing cadmium levels in soil (up to 57%), and cadmium uptake in plant root (up to 50%) and shoot (up to 70%) compared to the control. Chitosan-biochar rendered negative sites to bind Cd (II) ions effectively by cation exchange, precipitation, and surface complexation. The elevated soil pH also sturdily influenced the phytoavailability and mobility of cadmium. Higher soil pH may surge the soil’s negative charges, which may accelerate the cadmium immobilization via electrostatic attraction. Moreover, elevated soil pH expedited the generation of hydroxyl-bound species of cadmium^34^. In another study, Aoulad El Hadj et al.^27^ described that 2% goethite-doped biochar treatment could decrease arsenic uptake in *Oryza sativa* grains (up to 77%) compared to pristine biochar. The goethite-treated biochar enhanced the content of Fe oxide in soils, which stimulated the Fe-plaque formation in the root and ultimately reduced the arsenic content in *Oryza sativa* grains^150^. Several studies have also been carried out utilizing other kinds of biochar involving various engineering/modifying techniques such as ultraviolet activation, sulfur power, Al–Mg LDH, polyethyleneimine treatment, and CTAB doping to remediate metals/metalloid polluted soils^113^.

**Table 7:** Summary of various engineered/modified biochars and their immobilization efficiency for metals/metalloids in the soil system.

Biochar type	Pyrolysis temperature (°C)	Modification technique	Metalloids	Application rate/dose	Findings	References
Carrot pulp	550	Thiol-modification	Zinc (112 mg kg^−1^), Copper (29 mg kg^−1^)	4 and 8%	As compared to pristine biochar, thiourea-doped-biochar was more efficient in converting labile fractions to stable fractions of Zn/Cu in soil	^149^
Corncob	600	Magnesium chloride hexahydrate	Lead (3410 mg kg^−1^)	5%	MgO-coated biochar addition induced a significant 50% reduction in TCLP-leached Pb^2+^ in soil-washing residue	^150^
Peanut shell	600	CTAB	Chromium (1992 mg kg^−1^)	1,2 and 5%	Engineered-biochar exhibited higher Cr(VI) immobilization in soil, as showed by the substantial reductions in the bio-accessibility, (up to 97%), leachability (100%), and bioavailability (up to 92%) of Cr^6+^ than the pristine biochar	^151^
Rice straw	600	Red mud	Arsenic (122 mg kg^−1^)	1%	Modified biochar reduced (27%) of the Sodium bicarbonate-extractable arsenic, which is more efficient than using red mud (6%) and biochar (23%) alone	^27^
Wheat straw	500	Goethite	Arsenic (10 mg kg^−1^), Cadmium (10 mg kg^−1^)	2%	The arsenic and Cadmium content of Oryza sativa grains were reduced by 77% and 85%, respectively	^34^
Tea branch	500	Manganese ferrite	Cadmium (696 mg kg^−1^), Antimony (79 mg kg^−1^)	0.1, 1 and 2%	Ammonium nitrate -the extractable amount of antimony in soil reduced by 33 to 43% with Manganese ferrite-doped biochar treatments; the maximum reduction of Calcium chloride-extractable cadmium (up to 76%) was found at 2% additional dose	^62^
Rice straw	500	Thiol-modification	Lead (1182 mg kg^−1^), Cadmium (9.2 mg kg^−1^)	1 and 3%	Thiol-doped biochar decreased the soil-available lead by 8 to 11% and soil-available cadmium by 34 to 39%	^7^
Coconut shell	800	HCl and Ultrasonication	Cadmium (0.82 mg kg^−1^), Nickel (66 mg kg^−1^) Zinc (184 mg kg^−1^)	2.5 and 5%	5% engineered biochar addition resulted in soil-available zinc, nickel, and cadmium reduced by 30%, 57%, and 12%, respectively	^32^
Maize stalk	500	Polyethyleneimine	Cadmium (0.4 mg kg^−1^)	2600, 5200, and 13,000 kg ha^−1^	Polyethyleneimine-treated biochar decreased the cadmium uptake in the wheat by 40 to 80%; soil physicochemical characteristics such as CEC, pH, enzyme activities, and soil aggregates stability were increased after the application of polyethyleneimine-loaded	^152^
Maize stalk	350	Immobilization with *Citrobacte, Bacillus cereus* and *Bacillus subtilis* sp.	Uranium (29 mg kg^−1^) Cadmium (2 mg kg^−1^)	3%	The diethylenetriaminepentaacetic acid -extractable cadmium and cadmium in the soil reduced by 56 and 69%, respectively; bacteria-modified biochar decreased metal uptake hence stimulating celery growth	^84^
Fabric waste	600	Chitosan	Cadmium (20 mg kg^−1^)	5%	Chitosan-doped biochar application reduced the distribution of cadmium in roots (up to 54%), shoots (upto73%), and soil available cadmium (up to 58%) relative to control	^153^
Wheat straw	500	Bismuth nitrate pentahydrate	Arsenic (47 mg kg^−1^)	1,2 and 5%	The Bismuth nitrate pentahydrate-modified biochar reduced the (non)specifically adsorbed arsenic as the application rate raised, whereas pristine biochar caused the arsenic release	^154^
Animal manure	450	nZVI and chitosan	Chromium (100 mg kg^−1^)	5%	The engineered biochar exhibited simultaneous sorption of Cr^3+^ via precipitation and surface complexation and reduction of Cr^6+^ to Cr^3+^	^57^
Rice husk	550	Sulfur	Mercury (1000 mg kg^−1^)	5%	Compared to the control, 5% Sulfur-loaded biochar decreased freely available mercury in TCLP leachates by 99%	^38^
Corn straw	400	Immobilization with *Pseudomonas*	Copper (247 mg kg^−1^), Cadmium (56 mg kg^−1^)	5%	The addition of bacterial-modified biochar decreased the diethylenetriaminepentaacetic acid -extractable cadmium/copper	^19^
Corn straw	700	Ball milling	Lead (33 mg kg^−1^), Cadmium (1.28 mg kg^−1^)	2%	Soil-available lead and cadmium were reduced by 34% and 48%, respectively; Lead and cadmium uptake by corn was reduced	^30^
*Brassica napus*	600	Ultraviolet radiation	Cadmium (1.9 mg kg^−1^)	0.2,0.4 and 0.6%	With engineered biochar treatments, the Calcium chloride-extractable cadmium was decreased by 18 to 51%; and the uptake of cadmium in plant shoots was reduced by 67 to 82%	^32^
Bamboo	700	Al/Mg LDH	Uranium (33 mg kg^−1^)	10%	Modified biochar application decreased the cumulative loss (up to 53%) and leaching efficacy (54%) of uranium, relative to control	^32^
Kenaf bar	600	Ferrous sulfate heptahydrate	Cadmium (10 mg kg^−1^)	5%	Residual fractions of cadmium enhanced by 45% due to the Cd(II) complexation with iron hydroxides	^34^
Plant residues	650	Lead (736 mg kg^−1^) Cadmium (0.5 mg kg^−1^), Arsenic (141 mg kg^−1^)	Ferric chloride hexahydrate	3%	Fe-loaded biochar was suggested for remediation of Arsenic-polluted paddy soils while fresh biochar might be more appropriate for cadmium and lead remediation; bioavailability of lead, cadmium, and arsenic reacted differently to different water management regimes	^39^

Nonetheless, agricultural/farming soils are often concurrently polluted with several metalloids. Thus, many authors have also stated the influence of engineered/modified biochars in the immobilization of several metalloids in co-polluted soils. Mishra et al.^151^ synthesized MnFe_2_O_4_-engineered biochar produced from tea and investigated the effect of its addition with different rates such as 0.1, 1%, and 2% on the removal of Cd and Sb from the polluted soil. They found that MnFe_2_O_4_-engineered biochar treatment at 2% simultaneously reduced the amount of bioavailable Cd and Sb in soil by 76.0%, and 43.5%, respectively. Nonetheless, the raw biochar only decreased the bioavailable cadmium amount in soil (12%-33%). Goswami et al.^98^ assessed the effect of HCl and ultrasonication-activated coconut shells on the availability of Zn, Ni, and Cd in multi-metal polluted soils. After the incubation of 63 days, the addition of modified biochar at a 5% rate reduced the amount of bioavailable Zn, Ni, and Cd by 12%, and 57%, respectively relative to the un-modified biochar. The modified biochar contained abundant active surface functional groups such as C = O, -OH, and -COOH, and its addition enhanced the soil CEC and pH, which consequently reduced the metal ions mobility in the soil by cation exchange, surface complexation, and electrostatic attraction.

### Organic contaminants

In addition to metals/metalloids immobilization, biochars activated/modified by ball milling, CO_2_/steam activation, iron materials, oxidizing, bacteria loading, organic surfactants, LDH for the removal of several organic contaminants such as phenols, PAHs, plasticizer, antibiotics and pesticides in soil have been stated and are documented in (Table 8). Especially, a sulfidation- nZVI doped biochar created via^152^, the modified at a rate of 1% exhibited greater nitrobenzene degradation with an elimination rate (98%) within 24 h. They verified that the solubilization influence via biochar and reduction through FeSx were the leading mechanisms for the removal of nitrobenzene. Moreover, sulfidation-nZVI-doped biochar also had superior antioxidant capability and kept a great removal performance for nitrobenzene (up to 70%) after aging for 98 days, which advocated that this engineered biochar had great potential for field trials. Pan et al.^153^ produced biochar from olive residues at 400 °C, and KMnO_4_ (0.025 M) was employed as an oxidizing agent to synthesize the engineered biochar with great redox ability for the pentachlorophenol remediation. KMnO_4_-loaded biochar showed a higher remediation rate under anaerobic (2.4 μg PCP g^−1^ soil d^−1^) and aerobic (3.7 μg PCP g^−1^ soil d^−1^) conditions, which was much greater than that of fresh biochar^153^. Furthermore, *Bacillus siamensis* loaded biochar was produced to reduce dibutyl phthalate contamination in farming soils^84^. The bacterial-modified biochar could increase the biodegradation of dibutyl phthalate by simultaneously elevating the rate of degradation constant (from 0.20 to 0.24 d^−1^) and half-life (from 2.31 to 2.11 d^−1^). A substantial reduction of dibutyl phthalate uptake via leafy vegetables was also noticed, which could be accredited to the increased degradation/adsorption through *Bacillus siamensis* strain T_7_ and biochar^84^.

**Table 8:** Summary of various engineered/modified biochars and their immobilization efficiency for organic contaminants in the soil system.

Biochar type	Pyrolysis temperature (°C)	Modification agents/technique	Application rate	Organic contaminants	Immobilization performances	Main findings	References
Maize stalk	700	Sulfur-nZVI	0.25 and 1.5%	Nitrobenzene	0.72 mg g^−1^	The mass ratio of sulfur-nZVI and biochar was 3:1, the application rate was 0.5%, and 98% nitrobenzene removal was attained within 24 h	^152^
Rice husk	700	Rhamnolipid	2%	Petroleum	30 mg kg^−1^	The removal amount of total petroleum hydrocarbons for planted and un-planted soil and planted soil with rhamnolipid-treated biochar application were 8%, 19%, and 35%, respectively	^153^
Maize stalk	600	Acinetobacter-loaded and Ferric nitrate nonahydrate	0.1%	Atrazine	20 mg kg^−1^	Almost all the atrazine was degraded after treatment of engineered biochar, mainly owing to the Fe-loading boosted the microbial degradation capability as an electron transfer medium	^94^
Wheat straw	500	Ball milling	0.4%	Tetracycline	2.17 mg kg^−1^	96% removal of tetracycline was found after the application of ball-milled biochar owing to the degradation and adsorption mechanisms	^154^
Maize straw	650	KOH	1, 3, and 5%	Perfluorooctanoic acid	10 μg g^−1^	Application of KOH treated-biochar decreased the uptake (50%) and bioavailability (90%) of perfluorooctanoic acid in the polluted sediments	^63^
Corn straw	600	Fe/Mg-LDH	0.5%	Sulfamethoxazole	8 mg kg^−1^	Pot experiments exhibited that treated biochar could prompt urea-hydrogen peroxide to degrade sulfamethoxazole by 68%	^84^
Rice husk	500	*Bacillus siamensis*	3%	Dibutyl phthalate	100 μg g^−1^	Bacterial-inoculated biochar enhanced the biodegradation of Dibutyl phthalate in soil and reduced its uptake via leafy vegetables	^155^
Walnut shell	700	FeCl_3_ and Illite	0.2 and 4%	Metolachlor	10 to 120 mg L^−1^	Application of FeCl_3_ and Illite co-loaded biochar boosted the adsorption capability of soil (129 mg g^−1^) which was greater than control soil (72 mg g^−1^	^156^
Waste timber	900	CO_2_/steam activation	0.1 to 5%	Polyfluoroalkyl substances	1200 to 3800 μg kg^−1^	Activated biochars at a 5% rate strongly decreased leaching amounts of poly-fluoroalkyl by 98–100%	^157^
Maize straw	600	Fe(NO_3_)_3_ and KMnO_4_	0.5, 1, and 2%	Dibutyl phthalate	40 mg kg^−1^	The residual dibutyl phthalate in grains reduced by 28 to74% under engineered biochar treatments as the dose increased, while that of un-modified biochar treatment reduced by 6 to 51%, relative to the control	^158^
Basket willow	700	Microwaves	5%	PAHs	39.9 mg kg^−1^	The application of modified biochar decreased the dissolved PAH concentration in soils by (85%) relative to the unamended soils	^50^
Biogas residues	700	Potassium ferrate	1%	Benzo[a]pyrene	8.16 mg kg^−1^	The Fe-loaded biochar coupled with ammonium persulfate resulted in the degradation amount reaching 91% after 72 h in polluted soil	^67^
Sewage sludge	700	Rhamnolipid	2%	Petroleum	50,048 mg kg^−1^	Rhamnolipid-doped biochar exhibited superior capability for the degradation of petroleum than raw biochar (32 vs 21%)	^156^
Bur cucumber shoot	700	H_2_SO_4_	2%	Sulfamethazine	10 mg L^−1^	The loamy sand soil after H_2_SO_4_-treated biochar application exhibited a higher adsorption capacity for sulfamethazine, (182 mg kg^−1^)	^157^
Buffalo nutshell	500	Lanthanum ferrite	0.75 g L^−1^	PAHs	Total 61,586 ng g^−1^	With the application of lanthanum ferrite-loaded biochar, the total PAHs elimination reached (76%) which could be attributed to the interactions between the graphitized biochar network and surface oxygen species at lanthanum ferrite defective sites	^158^
Olive residue	400	Potassium permanganate	2.5%	Pentachlorophenol	2 to 30 μg g^−1^	The treated biochar was capable of achieving the maximum rates of remediation and great removal of extractable pentachlorophenol under both anaerobic and aerobic conditions	^159^
Palm branches	300	Chitosan	1%	Imazapyr and imazapic herbicides	500 mg L^−1^	For the removal of imazapyr and imazapic, the chitosan-doped biochar-amended soil respectively exhibited 84% and 73% removal efficacy, greater than control soil (8% and 50%)	^160^
Giant reed	500	Cupric nitrate	40%	Phenanthrene	0.013 mg L^−1^	Constructed wetlands with cupric nitrate-doped biochar eliminated (94%) phenanthrene	^161^

## Reusability of modified/engineered biochar

As with any biochar, consumed biochar gives a challenge, which requires suitable management. Depending on the kind of contaminants, and material cost, spent biochars are typically regenerated or become waste and incinerated or disposed of^154^. Consequently, adsorption pollutants are immobilized via the biochars, but the mechanism is typically reversible, particularly in the case of physical sorption. Therefore, the disposal of spent biochar with adsorbed contaminants in dumpsites takes the hazard of environmental pollution of groundwater, soil, and surface water, also creating additional costs^155^. Incineration of spent biochar contributes to the release of noxious gases, and ash creation possibly with risky elements, and needs money^156^. Regeneration and recovery of the spent biochars can decrease the cost and quantity of dumped waste. Chemical, microwave irradiation, and thermal regeneration with an inorganic or organic solvent regeneration are the techniques usually applied in biochar regeneration^157^. Nonetheless, in non-thermal approaches, occurring in the solution, the contaminant can desorb without degrading, thus there is a further problem with its elimination^60^. Therefore, the novel method for biochar regeneration is centered on catalytic oxidation such as the Fenton reaction leading to adsorbate degradation^158^. The efficiency of these approaches depends on several factors of which the most vital are the kind of biochar and interactions among adsorbate and adsorbent^159^. The regeneration through higher temperatures and chemicals affects the biochar characteristics such as porosity, structure of biochar, and functional groups and ultimately alters the catalytic functionality and sorption capacity of biochar and the resultant loss of their effectiveness^160^. This is particularly needed for the regeneration of engineered/modified biochar, which is typically characterized by the presence of some additives (catalytic compounds in doped biochar) and functional groups. Thus, it is substantial to have a case-by-case method depending on the kind of adsorbate-biochar system verified. Another alternative to spent biochar usage is the reuse of biochar with a bound contaminant in other several areas of life. Modified biochars used for the elimination of PO_4_^3−^ can be applied as a soil fertilizer and conditioner^161^. Studies show that biochars can be utilized in energy, e.g., an additive in biogas production and as a solid fuel^162^. Therefore, the use of exhausted biochars for these intentions seems to be possible. Nonetheless, there is no relevant information about this area, which should be examined more intensely in the future.

## Economic importance of biochar and its application

**Figure 6:**
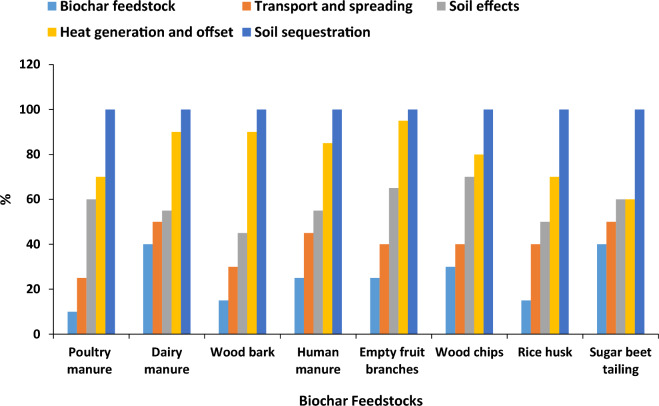
Economic benefits of biochars prepared from different feedstocks.

Pan et al.^163^ reported that the production cost of biochar from various biomasses has seldom been enlisted in previous literature. The cost of biochar depends on various factors including availability, collection, and transportation of raw biomass and scale, production technology, handling, and supply. Given biochar fabrication factors and transportation is the most significant parameter. They noticed the economic feasibility of making two kinds of biochar in three states and exposed that the net present cost of biochar increases with the reduction in movement of mobile pyrolysis unit. Figure 6 displays the economic paybacks of biochar utilization. Another significant factor of interest is the cost of labor in biochar fabrication, which varies globally as comparatively high in the USA and UK (about 5000 $(USD)/t) and less in India and the Philippines ($90/t)^112^. In general, biochar cost varies in several states of the globe, where, Shen et al.^164^ and Bhavani et al.^47^ figured $50 to 682.54/t of the biochar. Qiu et al.^74^ also noticed that the biochar was sold for about $200/t to farmers and suggested the price of biochar as ($300 to 500/t). Different kinds of biochar costs such as coconut coir, wood waste, and yard waste, $775/t, $329/t, $775/t, and $69/t respectively have been valued in previous studies^165,166^. Contrastingly, the cost of wood and yard waste biochar is greatly less than other materials such as (activated carbon $1500/t), and (zeolites $600/t) as well as other biochar ($50 to 2000/t), thus these could be utilized as a cost-effective and efficient material for nutrients restores from contaminated water^167^. Additionally, food waste feedstock is a profitable choice because of its low cost and easy availability. These characteristics limit the biochar fabrication cost from food waste to $50/t than that of conventional feedstocks ($2200/t)^168^. Biochar production is gaining attention because of its auspicious potential in the environment and energy. Gupta et al.^99^ presented the economic supposition results for the biochar production in Selangor at $532/year and the total income from the sale of biochar was $8012/year. Therefore, the net present cost for biochar fabrication, which was measured via the investment amount and the net income, exhibited positive outcomes of the economic feasibility of biochar^99^. Cost- effectuality of biochar fabrication depended on its retailing price, with a break-even of around $280/t for pyrolysis at 450 °C and approximately $220/t for pyrolysis at 300°C^169^. Panwar and Pawar^170^ revealed that yard waste was confirmed to be auspicious feedstock for biochar creation with a net margin of $16 and $69 for the low and high-income scenario of carbon dioxide equivalents (CO_2_e), such as horse and cattle manures. Thus, the production of biochar can be tempting if the proceeds of the above costs offset the economic prices of elevating, hauling, harvesting, and storing the feedstock, besides those of applying pyrolysis, transportation, and application of the biochar. As reported from the examination, the net margin of biochar production could be enhanced with low-cost feedstock and an auspicious processing approach^171^.

## Conclusions and future perspectives

Based on the above discussion, multiple modifications/engineering strategies have been employed to improve the physicochemical and biological properties of biochar. The multiple-functionalized biochars have been successfully utilized for the remediation of soil and aquatic systems contaminated by various pollutants. The specific attributes of multiple-modified biochar including the appearance of new functional groups, enhanced surface area, and greater electron transport capability are amongst the main factors affecting the remediation efficiency in multifaceted applications. Generally, functionalized biochar is an environmentally friendly catalyst/adsorbent that can be applied to address various environmental concerns. Nonetheless, some concerns remain unresolved and the following strategies need to be considered to attain a sustainable future for multiple-functionalized biochar in environmental applications. The superiority and remediation efficiency of multiple engineered/modified biochars is affected by the feedstock type, pyrolysis parameters, and modification techniques. A combined method following the modeling and experimental results should be employed to make standards for biochar fabrication, characterization as well and life cycle assessment (LCA) procedures. The presence of PFRs, PAHs, and heavy metals in biochar has been stated. Furthermore, some modification methods may introduce new hazardous substances. The stability and ecotoxicity of such potentially hazardous biochars should be assessed from an ecotoxicological perspective, including the toxic chemicals released over a long period. Engineered/modified biochars undergo long-term weathering via biotic and abiotic aging when exposed to environmental circumstances. Nonetheless, little knowledge is available about aged biochar remediation performance. Future research is required to investigate the stability of its decontamination potential with aging procedures and mechanisms influenced by various functionalization approaches. Chemical and physical modification techniques have often been employed to produce functionalized biochar. The production of functionalized biochar employing biological modification techniques involving microorganisms needs to be studied in detail about its significance in the decontamination of organic pollutants in soil and water systems. Advanced spectroscopic exploration techniques including XAFS (synchrotron-based X-ray absorption fine structure spectroscopy) and computational methods based on DFT (density functional theory) and MD (molecular dynamics) calculations should be explored to elucidate the remediation mechanisms for several contaminants. Machine learning and artificial intelligence should be applied as effective methods to enhance the development of functionalized biochar. Another advantage of modification is to attain easy removal of consumed biochar after contaminated-water treatment, for instance, magnetization, and the practical feasibility for recycling magnetic biochar demands to be examined at a pilot scale. Furthermore, there is scarce literature on the safe disposal of exhausted modified biochar after the sorption of toxic contaminants. Thus, related technology should be established to recover the exhausted functionalized biochar, i.e., use of specific solvents to effectively desorb the targeted pollutant. Additionally, non-renewable exhausted modified biochar should also be employed for energy creation from cost-effective and environmental perspectives. In the context of carbon neutrality worldwide, biochar as a carbon-negative technology has received wide attention. However, quantitative estimation methods of carbon sequestration by engineered biochar are missing, and the potential carbon sequestration value of engineered biochar has not been effectively verified and developed.

## Data Availability

All the data is available in the manuscript.
