# Author Correction: Recent trends and economic significance of modified/functionalized biochars for remediation of environmental pollutants

**DOI:** 10.1038/s41598-024-56882-w

**Published:** 2024-03-14

**Authors:** Ghulam Murtaza, Zeeshan Ahmed, Mohammad Valipour, Iftikhar Ali, Muhammad Usman, Rashid Iqbal, Usman Zulfqar, Muhammad Rizwan, Salman Mahmood, Abd Ullah, Muhammad Arslan, Muhammad Habib ur Rehman, Allah Ditta, Akash Tariq

**Affiliations:** 1 Faculty of Environmental Science and Engineering, Kunming University of Science and Technology, Kunming, 650500 China; 2 Xinjiang Institute of Ecology and Geography, Chinese Academy of Sciences, Urumqi, 830011 Xinjiang China; 3 Xinjiang Institute of Ecology and Geography, Cele National Station of Observation and Research for Desert‑Grassland Ecosystems, Chinese Academy of Sciences, Xinjiang, 848300 China; 4 Department of Engineering and Engineering Technology, Metropolitan State University of Denver, Denver, CO 80217 USA; 5 Center for Plant Science and Biodiversity, University of Swat, Charbagh, Pakistan; 6 Department of Botany, Government College University, Katcheri Road, Lahore, 54000 Punjab Pakistan; 7 School of Agriculture and Biology, Shanghai Jiao Tong University, Shanghai, China; 8 Department of Agronomy, Faculty of Agriculture and Environment, The Islamia University of Bahawalpur, Bahawalpur, Pakistan; 9 School of Energy Science and Engineering, Central South University, Changsha, 410011 China; 10 Faculty of Economics and Management, Southwest Forestry, Kunming, 650224 Yunnan China; 11 Institute of Crop Science and Resource Conservation (INRES), University of Bonn, Bonn, Germany; 12 Department of Seed Science and Technology, Institute of Plant Breeding and Biotechnology (IPBB), MNS-University of Agriculture, Multan, Pakistan; 13 Department of Environmental Sciences, Shaheed Benazir BhuttoUniversity SheringalDir(U), KPK, Upper Dir, Sheringal Pakistan; 14 School of Biological Sciences, The University of Western Australia, Perth, WA 6009 Australia

Correction to: *Scientifc Reports*, 10.1038/s41598-023-50623-1, published online 2 January 2024

In the original version of this Article, Usman Zulfiqar was incorrectly affiliated with 'School of Energy Science and Engineering, Central South University, Changsha 410011, China', Muhammad Rizwan was incorrectly affiliated with 'Faculty of Economics and Management, Southwest Forestry, Kunming, Yunnan 650224, China' and Salman Mahmood was incorrectly affiliated with 'Institute of Crop Science and Resource Conservation (INRES), University of Bonn, Bonn, Germany.'

Consequently, the correct affiliations are as follows:

Usman Zulfiqar:

Department of Agronomy, Faculty of Agriculture and Environment, The Islamia University of Bahawalpur, Bahawalpur, Pakistan.

Muhammad Rizwan:

School of Energy Science and Engineering, Central South University, Changsha 410011, China.

Salman Mahmood:

Faculty of Economics and Management, Southwest Forestry, Kunming, Yunnan 650224, China.

The original Article has been corrected.

